# Innate Immune Detection of Flagellin Positively and Negatively Regulates *Salmonella* Infection

**DOI:** 10.1371/journal.pone.0072047

**Published:** 2013-08-19

**Authors:** Marvin A. Lai, Ellen K. Quarles, Américo H. López-Yglesias, Xiaodan Zhao, Adeline M. Hajjar, Kelly D. Smith

**Affiliations:** 1 Department of Pathology, University of Washington, Seattle, Washington, United States of America; 2 Department of Comparative Medicine, University of Washington, Seattle, Washington, United States of America; University of Osnabrueck, Germany

## Abstract

*Salmonella enterica* serovar Typhimurium is a flagellated bacterium and one of the leading causes of gastroenteritis in humans. Bacterial flagellin is required for motility and also a prime target of the innate immune system. Innate immune recognition of flagellin is mediated by at least two independent pathways, TLR5 and Naip5-Naip6/NlrC4/Caspase-1. The functional significance of each of the two independent flagellin recognition systems for host defense against wild type *Salmonella* infection is complex, and innate immune detection of flagellin contributes to both protection and susceptibility. We hypothesized that efficient modulation of flagellin expression *in vivo* permits *Salmonella* to evade innate immune detection and limit the functional role of flagellin-specific host innate defenses. To test this hypothesis, we used *Salmonella* deficient in the anti-sigma factor *flgM*, which overproduce flagella and are attenuated *in vivo*. In this study we demonstrate that flagellin recognition by the innate immune system is responsible for the attenuation of *flgM^−^ S.* Typhimurium, and dissect the contribution of each flagellin recognition pathway to bacterial clearance and inflammation. We demonstrate that caspase-1 controls mucosal and systemic infection of *flgM^−^ S.* Typhimurium, and also limits intestinal inflammation and injury. In contrast, TLR5 paradoxically promotes bacterial colonization in the cecum and systemic infection, but attenuates intestinal inflammation. Our results indicate that *Salmonella* evasion of caspase-1 dependent flagellin recognition is critical for establishing infection and that evasion of TLR5 and caspase-1 dependent flagellin recognition helps *Salmonella* induce intestinal inflammation and establish a niche in the inflamed gut.

## Introduction

Innate immune recognition of *Salmonella* is mediated by evolutionarily conserved receptors [pattern recognition receptors (PRRs)] that are capable of sensing conserved bacterial structures [pathogen associated molecular patterns (PAMPs)] that promote inflammatory and immune responses [1]. PRRs recognize a wide range of microbial ligands such as bacterial DNA, cell wall components (peptidoglycan and LPS) and flagellin. In this study, we focus on *Salmonella* flagellin recognition by the innate immune system. Bacterial flagellin has been studied for decades due to its importance in motility. Flagellin based motility and chemotaxis are important for the induction of acute colitis, and to compete with other microbiota for nutrients in the inflamed gut [2]; [3]. Flagellin is also a target of the innate immune system. There are at least two flagellin recognition pathways in mammals: i) cytosolic flagellin is detected by Naip5-Naip6/NlrC4/Caspase-1 and ii) extracellular flagellin is detected by Toll-like receptor 5 (TLR5) [4]–[11].

Mice detect a conserved site in the carboxy-terminus of flagellin using the intracellular receptors Naip5 and Naip6, leading to Naip5/6 association with Nlrc4 and the activation of caspase-1 [4]–[6], [8], [9]. Nlrc4 contains a caspase activating region domain (CARD) and can associate with caspase-1 in the absence of Asc, promoting caspase-1 autocleavage and cell death [4], [12]. Association of the Nlrc4 inflammasome with Asc leads to efficient processing and secretion of IL-1B and IL-18 [13]. Caspase-1 protects mice against oral infection with *Salmonella*, which is dependent on IL-1B and IL-18 production and mediated by the Nlrc4 and Nlrp3 inflammasomes [13]–[15]. The Nlrc4 inflammasome also controls intraperitoneal infection of mice from *Salmonella* that overexpress flagellin, where Nlrc4–mediated protection is dependent on pyroptosis and independent of IL-1B and IL-18 [16]. The relevance of caspase-1 dependent flagellin detection during *Salmonella*-induced enterocolitis has not been fully elucidated.

TLR5 recognizes a conserved surface in the D1 domain of monomeric flagellin, and activates NF-kB dependent signaling in epithelial and immune cells [7], [10], [11], [17], [18]. TLR5 stimulation has been associated with release of inflammatory mediators, such as CCL20, a strong DC chemoattractant; IL-6 and IL-12/IL23p40; Reg3γ, an antimicrobial peptide; IL-8-like chemokines; and IL-10, an immunosuppresor [19]–[22]. Alexopoulo and colleagues found that TLR5 protected mice against *Salmonella* infection, but that this function was largely masked by TLR4 [23]. Akira and colleagues demonstrated that TLR5 recognition of flagellin promoted *Salmonella* infection and systemic spread [24]. Gewirtz and colleagues demonstrated that loss of TLR5 led to an increased basal inflammatory state and non-specific resistance to *Salmonella* infection, suggesting that TLR5 interactions with the intestinal microbiota may promote *Salmonella* infection [25], [26]. Thus the role of TLR5 in mucosal *Salmonella* infection is complex, and involves both homeostatic interactions with gut commensals that influence microbial composition and immune status, and detection of flagellated pathogens.

Flagellar assembly is under complex and tight regulation, with multiple signals that converge on the operon for the master regulator flhDC, as well as additional transcriptional, translational and post-translational mechanisms that control the expression of class II and III genes, and the assembly of the flagellum [27]. FlgM inhibits FliA, the sigma factor required for the transcription of class III flagellar genes [27]. Because flagellar assembly is costly, complex regulation may help conserve energy for growth in certain environmental conditions [28], [29]. *Salmonella* selectively represses flagellin expression in systemic sites during infection, suggesting that active evasion of immune surveillance may also be critical for *Salmonella* to establish systemic infection [2], [30], [31]. During infection *Salmonella* has several mechanisms to sense the host environment and regulate gene expression. Some of these sensors, such as PhoPQ and ClpXP, down regulate flagellin synthesis [32]–[34]. Functional FlgM is also required to silence flagellin expression *in vivo*, and bacteria with deletions in *flgM* are attenuated during infection of mice in a flagellin-dependent manner [35]. We hypothesized that FlgM-dependent flagellin silencing is critical for *Salmonella* to evade innate immune detection, and that either TLR5 or caspase-1 are responsible for the attenuation of *flgM^−^ Salmonella*.

Our studies demonstrate that TLR5 and caspase-1 have modest roles in host defense against wild type *Salmonella* infection, which is largely due to efficient FlgM-dependent silencing of flagellin expression. When flagellin production is dysregulated by deletion of *flgM*, TLR5 paradoxically limits cecal inflammation and promotes cecal colonization, but does not alter systemic dissemination. In contrast, *flgM^−^ Salmonella* are efficiently cleared by the innate immune system in a caspase-1 dependent manner. In addition, caspase-1 is critical for limiting mucosal inflammation, and modulating the production of several cytokines and chemokines that may play cytoprotective roles in *Salmonella*-induced enterocolitis. Thus caspase-1 has ideal properties for innate immune defense at mucosal surfaces, and simultaneously limits bacterial dissemination and mucosal inflammation.

## Methods

### Ethics Statement

This study was carried out in strict accordance with the recommendations in the Guide for the Care and Use of Laboratory Animals of the National Institutes of Health. All protocols were approved by the Institutional. Animal Care and Use Committee of the University of Washington (protocol: 4031-01, Macrophage Biology).

### Bacterial Strains

The experiments were performed using wildtype *Salmonella* enterica serovar Typhimurium strain SL1344 (WT) (from Brad Cookson, University of Washington). The flagellin-deficient (*fliC^−/^fljB^−^*) strain in this same background was also a gift from Brad Cookson (University of Washington). A *flgM* deletion mutant was made in SL1344 using lambda-red technology [36]. The deletion was confirmed by PCR, and also transferred to the *fliC^−/^fljB^−^* mutant using P22 phage [37]. The deletion of *flgM* was once again confirmed by PCR. A constitutive GFP expressing plasmid (pDW5: ptetA::gfp; *gfp* downstream of *tetA* promoter in pBR322); reference PMID: 16803592) was introduced in *Salmonella* for fluorescent microscopy studies, the plasmid was a gift from Brad Cookson (University of Washington). Bacteria were grown in Luria broth at 37°C with aeration.

### Mouse Infection

WT C57BL/6J (B6) mice were purchased from Jackson Labs and housed or bred in our facilities at the University of Washington. Caspase 1^−/−^ [38] and Toll-like receptor 5 deficient mice (*TLR5^−/−^*) [24] on a B6 background were bred in our specific pathogen free (SPF) animal facilities. Animals were housed individually or in groups of up to five animals under standard barrier conditions in individually ventilated cages. 8–14 weeks old mice were used for infections throughout this study. One day before infection, food was withdrawn 4 h before oral gavage with 20 mg of streptomycin in 0.1 ml of PBS [39]. Afterwards, animals were supplied food ad libitum. At 20 h after streptomycin treatment, food was withdrawn again for 4 h before the mice were orally infected with 1000 CFU *S*. Typhimurium (delivered in 0.1 ml of PBS by gavage) or sterile PBS (control). The inoculum containing *Salmonella* was prepared by back-diluting an overnight culture 1∶50 in LB +50 µg/ml of streptomycin. After 4 hours, the concentration of bacteria was measured and diluted in cold PBS to a coentration of 1×10^4^ *CFU/ml*, and CFU of the inoculum was verified by plating on LB agar plates with 50 µg/ml streptomycin. Food was replaced immediately after gavage. Five days post-infection, mice were sacrificed by CO_2_ asphyxiation, and blood and tissue (intestine, mesenteric lymph node, spleen, and liver) were promptly removed. Tissue samples from cecum and spleen were stored at −80°C for RNA extraction. Additional samples from all tissues were fixed in 10% formaldehyde, or snap frozen in OCT medium for histopathological analysis. For some infections, cecal samples were also placed in Karnovsky’ fixative for ultrastructural studies. Bacterial burden was assessed by weighing and homogenizing the tissues in PBS with 0.025% Triton X-100, and plating dilutions of the samples on MacConkey agar plates with streptomycin (50 ug/ml). For the homogenizing step, the ceca were scraped and blotted to remove fecal content.

### Competitive Index Infection

The procedure for competitive index experiments followed the mouse infection protocol described above with the following exceptions: 1) the inoculum prepared contained equal numbers (1000 CFU) of WT and *flgM^−^* Salmonella, the inoculum was verified by plating bacteria on antibiotic specific LB plates (flgM^−^ Salmonella were kanamycin and streptomycin resistant (kan^r^-str^r^) and wildtype Salmonella were streptomycin resistant only (kan^s^-str^r^); 2) dilutions of the homogenized tissues (liver, spleen, MLN, and cecum) were plated on both kanamycin/streptomycin (50 µg/ml each) and streptomycin (50 µg/ml) MacConkey agar plates; and 3) the competitive index (C.I.) of flgM−/WT *Salmonella* was determined by the following equation:

C.I. = CFU kan^r^−str^r^/(CFU kan^s^−str^r^ − CFU kan^r^−str^r^).

### Quantitative Histologic Assessment of Inflammation in Cecum, Small Intestine, Liver, Spleen and MLN

The formalin-fixed tissue was embedded in paraffin using standard protocols. 4 µm thick sections were stained with hematoxylin and eosin using standard procedures. A blinded pathologist (KDS) examined the slides and scored them according to the following criteria. Scores were assigned for changes to the cecum as follows: **submucosal expansion (S)** - 0 = no significant change, 1 = <25% of the wall, 2 = 25–50% of the wall, 3 = >50% of the wall; **mucosal neutrophilic infiltrate (M)** - 0 = no significant infiltrate, 1 = mild neutrophilic inflammation, 2 = moderate neutrophilic inflammation, 3 = severe neutrophilic inflammation; **lymphoplasmacytosis (L)** - 0 = no significant infiltrate, 1 = focal infiltrates (mild), 2 = multifocal infiltrates (moderate), 3 = extensive infiltrates involving mucosa and submucosa (severe); **goblet cells (G)** - 0 = >28/HPF, 1 = 11–28/HPF, 2 = 1–10/HPF, 3 = <1/HPF; **epithelial integrity (E)** - 0 = no significant change, 1 = desquamation (notable shedding of epithelial cells into the lumen), 2 = erosion (loss of epithelium with retention of architecture), 3 = ulceration (destruction of lamina propria).

### Western Blot

Single colonies were grown overnight at 37°C with constant shaking, back diluted the next day 1∶50 and grown under similar conditions for 3 hrs. Equal numbers of cells were pelleted, the pellets and the supernatants were separated by SDS-PAGE, and transferred to Immobilon-P filters (Source, San Diego, CA). Filters were blocked with 5% non-fat milk, and Antibodies specific for *Salmonella* flagellin were used to detect the protein on the blot. The primary anti-FliC antibody was a monoclonal purified anti-FliC (Biolegend) at a concentration of 1∶1000 diluted in PBS (Invitrogen) plus 1% BSA (SIGMA). The secondary antibody was a peroxidase-conjugated affinipure Goat anti-mouse IgG (H+L) (Jackson laboratories) at a concentration of 1∶1000 diluted in PBS plus 1% BSA.

### Motility Assay

Motility was tested by using motility plates (LB with 0.3% agar). The radius of growth was measured at different time points during culture at 37°C.

### Macrophage Cytotoxicity Assay

Thioglycollate elicited peritoneal macrophages were plated in a 96-well plate at a concentration of 5×10^5^ macrophages/well in RPMI 1640 medium with L-glutamine, 10% fetal bovine serum. *S.* Typhimurium was grown overnight in LB and backdiluted the next day 1∶50 in LB medium and grown for 3–4 hrs. The bacteria was centrifuged (1000 rpm, 5 minutes) and the pellet resuspended to the final desired concentration of bacteria/ml. Macrophages were infected with the desired multiplicity of infection, centrifuged at 250×g for 5 minutes (to ensure infection of macrophages), and the infection was allowed to progress for an hour. Gentamicin (50 ug/ml) was added after an hour to kill extracellular bacteria. After an additional hour the supernatants were removed, and cytotoxicity was measured using Cytotox 96 kit (Promega).

### Real-time PCR Analysis of Gene Expression in Cecum

Tissue was collected immediately post-euthanization and stored frozen at −80°C. The tissue was later homogenized in Trizol reagent (Invitrogen, Carlsbad, CA), and total RNA was isolated according to manufacturer’s procedure. Genomic DNA was removed with DNA-*free* ^™^ (Ambion, Austin, TX), and cDNA was synthesized from 5 ug total RNA using Superscript III (Invitrogen, Carlsbad, CA). Multiplex real-time PCR was performed using primer probes sets for CCL20, CSCL1, CXCL2, IFNG, IL-12A, IL-12B, IL-17, IL-1B, IL-22, IL-23A, IL27, IL-6, LCN2, RG3G, TNF (all FAM labeled); the GAPDH control (VIC-labeled) was included in each reaction to normalize results. All reagents were from Applied Biosystems, and samples were analyzed on an ABI Prism 7900 using the fast protocol and reagents.

### Measurements of Serum Cytokines by ELISA

Blood was collected from euthanized animals by cardiac puncture, and the serum was collected, centrifuged at 7000 g for 10 minutes and frozen at −20°C. ELISAs for TNF-α, IL-12, IL-6 and IL-1B were performed on serum samples according to manufacturer’s protocol (R&D systems, Minneapolis, MN).

### Fluorescent Staining

Frozen tissue blocks collected from mice infected with *Salmonella* containing a GFP plasmid were cut in 4 µm sections using a cryostat. Sections were fixed using acetone (-20°C, Fisher Chemicals, Hanover Park, Il), washed with PBS (4°C) and blocked with blocking buster™ (Innovex Bioscience, Redmond, CA) for 10 minutes at room temperature. The sections were washed with cold PBS and exposed to the F4/80 antibody (Rat anti-mouse, Serotec Inc, Raleigh, NC; diluted in PBS +1% BSA 1∶200) or TROMA-1 antibody (Rat anti-mouse, Developmental Studies Hybridoma Bank at the University of Iowa. Iowa city, IA; diluted in PBS +1% BSA 1∶200) for 2 hours at room temperature. The sections were then washed with cold PBS and an Alexa fluor 594 secondary antibody (donkey anti-rat, Invitrogen, Carlsbad, CA) was used at a concentration of 1∶200 for 2 hrs. The sections were then washed with cold PBS and Vectashield™ mounting buffer with DAPI was added to cover all tissue (Vector labs, Burlingame, CA). *Salmonella* associated cells were quantified manually by counting the number of F4/80+ positive cells that co-localized with GFP-positive Salmonella; at least 100 cells were counted to determine the percentage of *Salmonella* carrying cells. The number of GFP-positive Salmonella associated with each F4/80+ cells was determined by counting the number of bacteria within the F4/80+ staining region for each infected cell (in WT cecum tissue, we counted the number of intracellular bacteria in 33 cells, in caspase-1−/− cecum tissue, we counted 48 cells).

### Electron Microscopy (EM)

During dissection, the ceca of WT and caspase-1−/− mice orally infected with *flgM^−^* was fixed in 1/2 strength Karnovsky’s, post fixed in osmium tetroxide and then embedded in Eponate-12 by standard EM procedures. Tissue processing for EM by the Pathology Research Service at the University of Washington, Seattle. For detailed instructions please refer to [40], [41].

### Statistics

Significance was obtained by using the software GraphPad Prism (San Diego, CA). The Mann-Whitney test was used for all data where significance is shown. In all graphs, significance was established and represented using the following system: * = p<0.05, ** = p<0.01, *** = p<0.001. One way ANOVA was used when comparing three groups or more, using either the Dunn’s (non-Guassian) or Bonferroni’s (Guassian) multiple comparisons test. Statistical significance was represented as above.

## Results

### TLR5 Promotes and Caspase-1 limits *Salmonella* Infection in Streptomycin-pretreated Mice

**Figure 1:**
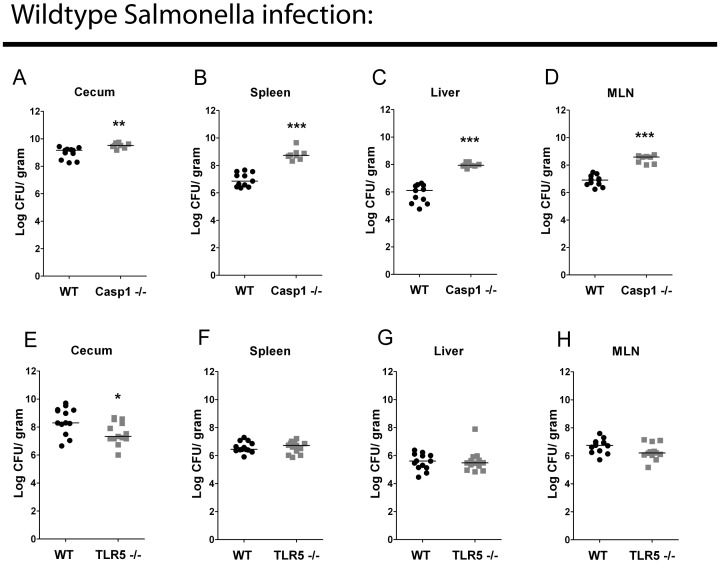
Wildtype *Salmonella* efficiently evades flagellin detection during acute mucosal infection in streptomycin-pretreated mice. Bacterial burden of WT C57BL/6 (n = 11) and caspase-1−/− (n = 8) mice infected with 1000 cfu WT SL1344 *Salmonella* in the cecum (A), spleen (B), liver (C), MLN (D). Bacterial burden of WT C57BL/6 (n = 12) and TLR5−/− (n = 13) mice infected with 1000 cfu wildtype *Salmonella* in the cecum (E), spleen (F), liver (G), MLN (H). Figures A–D are the combined data from two independent experiments. Figures E–H are the combined data from three independent experiments. Mann-Whitney test. * = p<0.05. ** = p<0.01. *** = p<0.001.

Flagellin recognition by the innate immune system is dependent on two pathways, the TLR5 pathway and a Naip5–6/Nlrc4/caspase-1 pathway. In order to establish the importance of each pathway, we infected *TLR5^−/−^* mice or *Casp1* ^−/−^ mice with WT *Salmonella*. Caspase-1 protects against systemic dissemination of WT *Salmonella* during oral infection in mice (Fig. 1 A–D). WT *Salmonella* colonized the systemic organs of *TLR5^−/−^* and WT C57BL/6 mice equally well, suggesting that WT *Salmonella* efficiently evade TLR5 recognition of flagellin (Fig. 1 E–H). Paradoxically, the cecal bacterial load was decreased in *TLR5^−/−^* mice, indicating a role for TLR5 in the regulating cecal colonization by *Salmonella* (Fig. 1 E).

### Caspase-1 Mediated Protection is Dependent on *Salmonella* Flagellin

**Figure 2:**
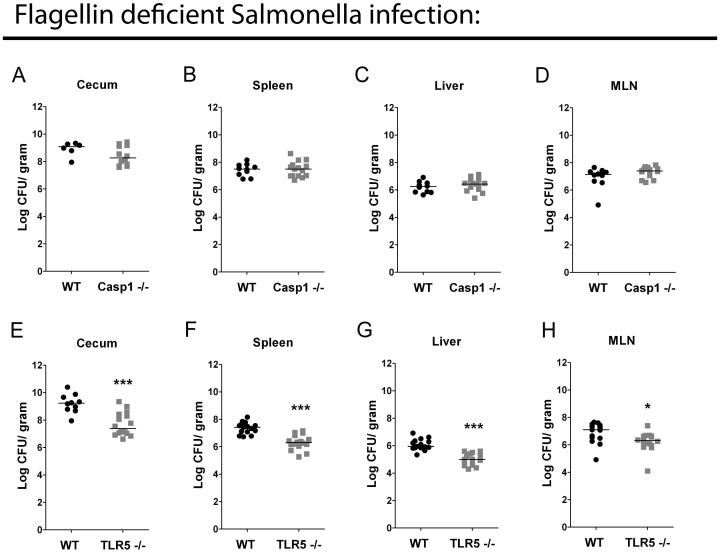
Flagellin detection accounts for the caspase-1 increased susceptibility during acute mucosal infection in streptomycin-pretreated mice. Bacterial burden of WT C57BL/6 (n = 10) and caspase-1−/− (n = 16) mice infected with 1000 cfu flagellin*^−^ Salmonella* in the cecum (A), spleen (B), liver (C), MLN (D). Bacterial burden WT C57BL/6 (n = 15) and TLR5−/− (n = 16) mice infected with 1000 cfu flagellin*^−^ Salmonella* in the cecum (E), spleen (F), liver (G), MLN (H). Figures A–D are the combined data from two independent experiments. Figures E–H are the combined data from three independent experiments. Mann-Whitney test * = p<0.05. *** = p<0.001.

We next infected *Casp1−/−* mice with flagellin-deficient *Salmonella* to determine if flagellin is needed for enhanced virulence in *Casp1^−/−^* mice. Flagellin-deficient *Salmonella* colonized the cecum, spleen, liver and MLN of *Casp1^−/−^* and WT mice equally well, indicating that caspase-1 mediated control of *Salmonella* infection is dependent on flagellin expression by *Salmonella* (Fig. 2 A–D).

### TLR5 Promotes Infection Independent of *Salmonella* Flagellin

To determine if flagellin is needed for the increased cecal colonization in WT relative to *TLR5^−/−^* mice, we infected these mice with flagellin-deficient bacteria. Flagellin-deficient *Salmonella* were even more attenuated than WT bacteria in the *TLR5^−/−^* mice, and fewer bacteria were recovered from all organs (Fig. 2 E–H). Thus TLR5 promotes *Salmonella* infection independently of flagellin expression by *Salmonella*.

### *In vitro* Characterization of *flgM-*deficient *Salmonella*

**Figure 3:**
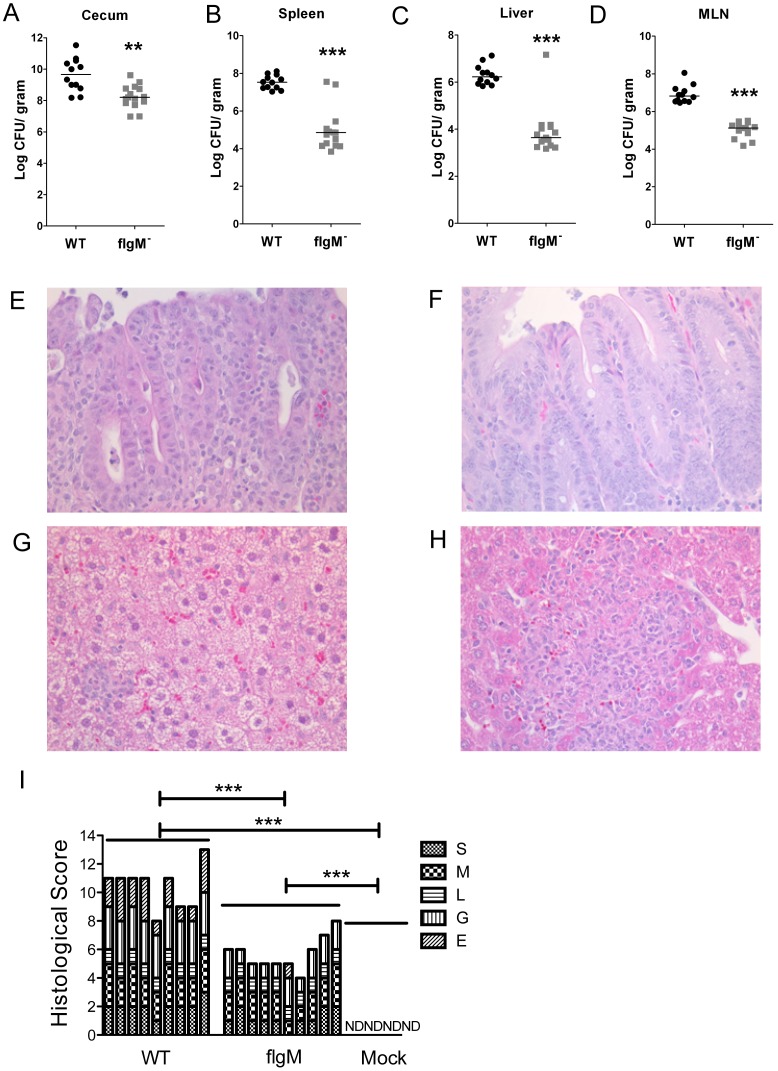
*flgM^−^ Salmonella* has an attenuated *in vivo* phenotype. C57BL/6 mice were infected with 1000 CFU of either WT SL1344 (n = 12) or *flgM^−^* (n = 15) *Salmonella*. (A) Bacterial burden in the cecum. (B) Spleen. (C) Liver. (D) MLN. Representative histology of the cecum (E+F) and liver (G+H) infected with WT (E+G) or *flgM^−^ Salmonella* (F+H). (I) Histological scores for changes in the cecum. Submucosal expansion (S); mucosal neutrophilic infiltrate (M); lymphoplasmacytosis (L); goblet cells (G); epithelial integrity (E). Liver images (400X magnification), cecal images (200X magnification). Figures A–D are the combined data from three independent experiments. Mann-Whitney test. ** = p<0.01, *** = p<0.001.

O’Brien and colleagues determined that *flgM* is required for *Salmonella* virulence [35], [42], [43]. FlgM hypersecretion due to a mutation in *fliD* is also associated with decreased virulence [44]. The loss of virulence in FlgM defective *Salmonella* requires *fliA* and *fliC* [35], indicating that FlgM inhibition of FliA-dependent flagellin expression is necessary for virulence. To confirm these previous studies and extend these findings to *Salmonella* induced enterocolitis, we deleted the entire *flgM* gene from *S.* Typhimurium SL1344 by homologous recombination (see methods). The *flgM* deletion (referred to as *flgM^−^* throughout this paper) secreted more flagellin *in vitro* and had more cell-associated flagellin (Fig. S3 A), but did not affect growth *in vitro* (Fig. S3 B) or motility (Fig. S3 C). In addition, *flgM^−^* and wild type *Salmonella* killed macrophages equally well, and this was largely flagellin-dependent (Fig. 3 D). Finally, *flgM^−^ Salmonella* produced more TLR5 stimulatory activity than WT *Salmonella*, consistent with greater flagellin production by this mutant (Fig. S3 E).

### Deletion of *flgM* Attenuates *S.* Typhimurium *in vivo*

Mice infected with *flgM^−^* had substantially and significantly less bacteria in their tissues, which was modest in the cecum (Fig. 3 A), and more pronounced in the spleen, MLN and the liver (Fig. 3 B–D). The ceca of WT and *flgM^−^* infected mice were thick walled, pale and small (Fig. S1 A). Histologically, WT *Salmonella* infected mice exhibited prominent epithelial injury, edema, and leukocytic infiltration (Fig. 3 E). In contrast, the ceca of *flgM^−^* infected mice showed moderate inflammation, characterized by edema and moderate leukocyte infiltration, with good preservation of the epithelium (Fig. 3 F). Quantification of cecal inflammation demonstrated that, *flgM^−^ Salmonella* induced less inflammation relative to WT (Fig. 3 I). We measured gene expression in the cecal tissue from these mice. Compared to mock-infected animals both WT and *flgM^−^* infected mice demonstrated significantly increased expression of Cxcl1, Cxcl2, IL-1B, Reg3g, Lcn2 and TNF, and the *flgM^−^* infected mice also had increased levels of Ccl20, IFN-γ and IL-6 (Fig. S2 A). Mice infected with *flgM^−^ Salmonella* had significantly increased Reg3g expression compared to WT infected mice (Fig. S2 A).

Systemically, *flgM^−^* infected mice had discrete foci of inflammation in the liver and spleen with no significant histologic evidence of hepatotoxicity (Fig. 3 H and data not shown), while WT infected mice exhibited severe hepatotoxicity, characterized by prominent vesicular change within the hepatocyte cytoplasm, and overall less inflammation (Fig. 3 G). *Salmonella* did not colocalize with hepatocytes suggesting that liver damage was indirect and not due to direct infection of hepatocytes (data not shown). Thus we hypothesized that the prominent hepatotoxicity was the result of a cytokine storm in the setting of systemic infection and sepsis [45]. Measurement of serum cytokines confirmed that WT infected mice had higher levels of pro-inflammatory cytokines such as TNF-α and IL-6 (Fig. S4 A, S4 C respectively). Serum levels of IL-1B were similar in all infected groups (Fig. S4 B). In contrast IL-12/23p40 was higher in *flgM^−^* infected mice (Fig. S4 D).

### The Attenuated Phenotype of *flgM^−^* is Dependent on Flagellin

**Figure 4:**
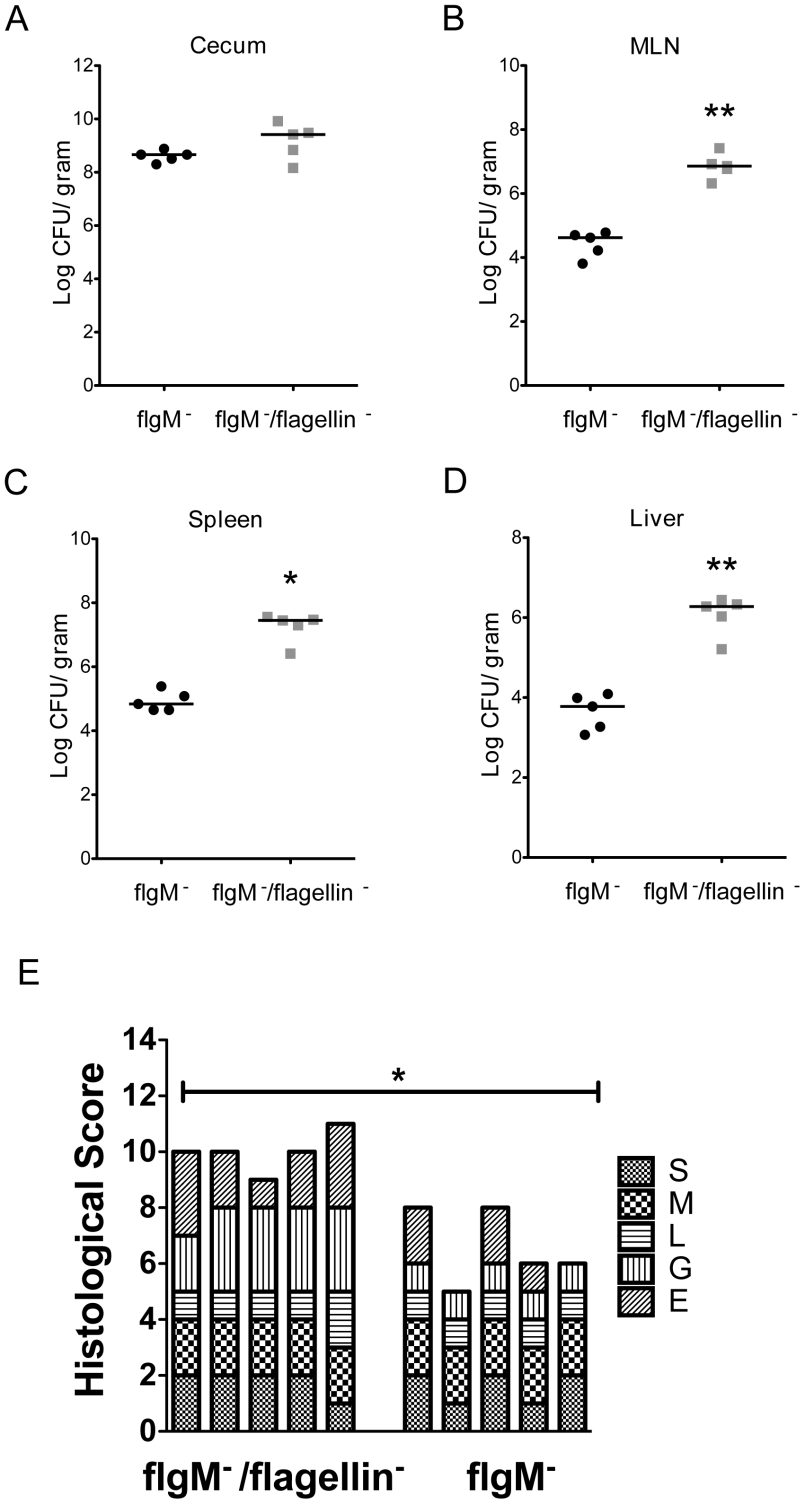
*flgM^−^ Salmonella* attenuated phenotype is dependent on flagellin. C57BL/6 WT mice were infected with 1000 CFU of either *flgM^−/^flagellin^−^* (n = 5) or *flgM^−^* (n = 5) *Salmonella*. Bacterial burden in the cecum (A). MLN (B). Spleen (C). Liver (D). (E) Histological scores for changes in the cecum in cecal pathology as described in Fig. 4. Figures A–D represent data from one experiment (*flgM^−^* n = 5; *flgM^−/^flagellin^−^* n = 5). Mann Whitney test. * = p<0.05, ** = p<0.01, *** = p<0.001.

To verify that the decreased bacterial burden and inflammation in *flgM^−^* infected mice was dependent on flagellin, we deleted the flagellin genes, *fljB* and *fliC*, in the *flgM^−^* bacteria. In the cecum the bacterial burden of *flgM*/flagellin-deficient *Salmonella* was comparable to that of *flgM^−^* (Fig. 4 A). The failure of the flgM/flagellin-deficient *Salmonella* to regain the modest growth advantage in the cecum may be due to loss of motility [2]. Deletion of the flagellin genes restored virulence to *flgM^−^* bacteria as evidenced by increased CFU in the MLN, liver and spleen (Fig. 4 B–D). The *flgM*/flagellin-deficient strain also induced hepatotoxic changes as seen with WT *Salmonella* (data not shown). Deletion of the flagellin genes restored inflammation and injury in the cecal mucosa, which was confirmed by histologic quantification, demonstrating decreased goblet cells and loss of epithelial integrity (Fig. 4 E and fig. S1 B). Thus loss of *flgM* results in flagellin-dependent attenuation of *S.* Typhimurium, which is manifest as decreased mucosal inflammation, mucosal injury, hepatotoxicity and systemic dissemination of bacteria.

### Caspase-1 is Required for Attenuation of *flgM*-deficient *Salmonella*

**Figure 5:**
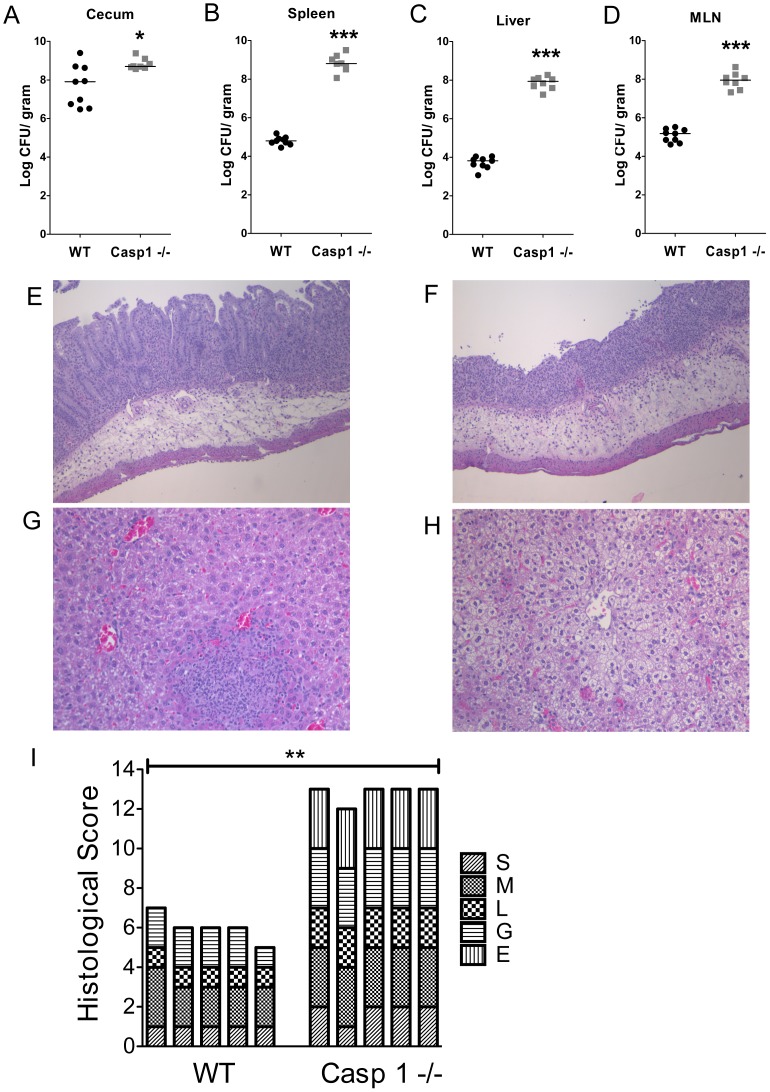
Caspase-1 is required for attenuation of *flgM*-deficient *Salmonella*. C57BL/6 WT (n = 9) and caspase 1−/− (n = 8) mice were infected with 1000 CFU *flgM^−^ Salmonella.* Bacterial burden in the cecum (A). Spleen (B). Liver (C). MLN (D). Representative histology of the cecum of WT mice (E) or caspase-1−/− (F), and liver of WT mice (G) or caspase-1−/− (H). (I) Histological scores for changes in the cecal pathology as described in Fig. 4. Figures A–D represent data from two independent experiments. Liver images (200X magnification), cecal images (100X magnification). Mann-Whitney test, * = p<0.05, ** = p<0.01, *** = p<0.001.

We next investigated the role of caspase-1 in flagellin-dependent attenuation of *flgM^−^ Salmonella*. The bacterial burden in the cecum of *Casp1^−/−^* mice was significantly increased compared to WT C57BL/6 mice (Fig. 5 A), and differences were even greater in the spleen, liver and MLN (Fig. 5 B, C, D respectively). Hepatotoxic changes were also observed in the liver of *Casp1* ^−/−^ mice, while WT mice exhibited only mild changes characterized predominantly by discrete foci inflammation (Fig. 5 G, H). Once again, the hepatotoxic changes correlated with higher serum levels of TNF (Fig. S5 A). We also observed higher serum levels of IL-1B and IL-6 and lower IL12/IL23p40 in *Casp1^−/−^* mice (Fig. S5 B–D). The ceca of all infected mice were thick walled, small and pale (Fig. S1 C). However, compared to WT mice, *Casp1* ^−/−^ mice had more severe inflammation and injury in the cecum (Fig. 3 E, F), which even exceeded the histologic changes seen in WT mice infected with WT *Salmonella* (Fig. 3 I) or *flgM*/flagellin-deficient *Salmonella* (Fig. 4 E). To further verify the importance of caspase-1 in the attenuation of *flgM*-deficient *Salmonella*, we performed competitive infection studies where mice were orally infected with equal numbers of WT and *flgM*-deficient *Salmonella*. The WT *Salmonella* outcompeted *flgM*-deficient *Salmonella* in the cecum, liver, spleen and MLN in WT mice, but not *Casp1* ^−/−^ mice (Fig. S6). These data suggest that caspase-1 dependent flagellin recognition is primarily responsible for the attenuated phenotype of *flgM^−^ Salmonella*.

### *Casp1* ^−/−^ Mice have Altered Gene Expression in the Cecum

*Casp1* ^−/−^ mice infected with *flgM^−^ Salmonella* had higher bacterial counts in the cecum and higher histologic inflammatory scores compared to WT *Salmonella* infections in WT mice (Fig. 5 I). To assess the importance of caspase-1 in mucosal immunity, we analyzed gene expression in ceca of WT and Casp1−/− mice infected with *flgM^−^*. Several genes that are involved in mucosal protection and immunity, such as Cxcl2 and IL-12A, retain levels of expression that are comparable to those observed in WT C57BL/6 mice and Lcn2, a gene that has been associated with gut inflammation [46], was detected at higher levels in *Casp1* ^−/−^ mice (Fig. S2 C). Paradoxically, *Casp1* ^−/−^ mice had significantly lower levels of Ccl20, Cxcl1, and Reg3γ (Fig. S2 C). This finding suggests that during *Salmonella*-induced enterocolitis caspase-1 selectively affects the expression of genes, of which some may be important for cytoprotection. Because Ccl20, Cxcl1 and Reg3γ are all known to be expressed by epithelial cells, and their expression is decreased in *flgM-*infected *Casp1^−/−^* mice but not *flgM*/flagellin- infected WT mice (Fig. S2 C), caspase-1 may also regulate cecal inflammatory responses in a flagellin-independent manner.

### Caspase-1 Limits Intracellular Growth of *Salmonella*

**Figure 6:**
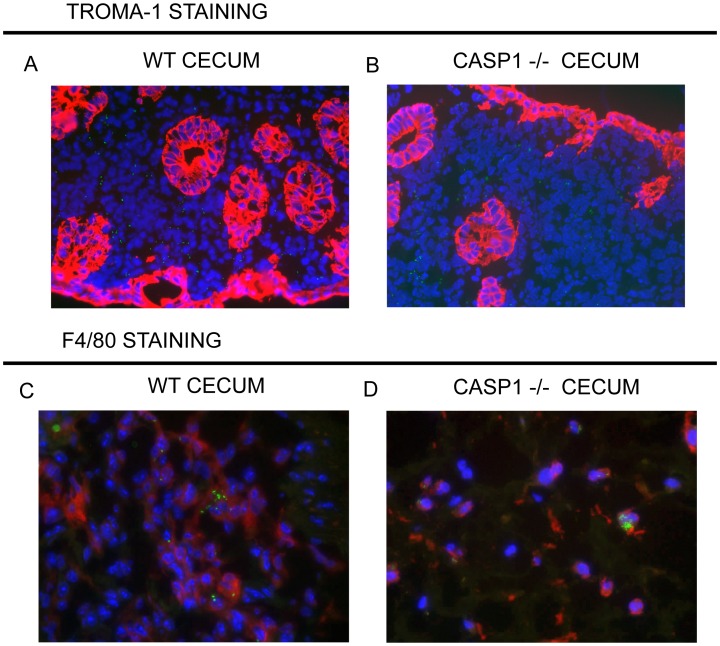
*Salmonella* reside within F4/80+ cells the cecum. C57BL/6 WT (n = 5) (A+C) or Caspase-1−/− (n = 5) (B+D) mice were infected with 1000 cfu of *flgM^−^ Salmonella* containing a stable GFP expressing plasmid. Intestinal epithelial cells were stained using TROMA (A+B), and phagocytes were stained using F4/80 (C+D) antibody. Red = TROMA-1 (A, B) and F4/80 (C, D); green = GFP (*Salmonella*). Shown are representative images from 10 mice examined.

Using fluorescent microscopy we studied the location of *Salmonella* in the cecum and the contribution of caspase-1 *in vivo*. In the cecum, *Salmonella* does not reside in intestinal epithelial cells of WT mice as demonstrated by TROMA-1 staining and only rarely present in epithelial cells of *Casp1* ^−/−^ mice (Fig. 6 A, B respectively). This observation was confirmed by electron microscopy, where no bacteria were seen in epithelial cells of WT mice, and only few cytoplasmic bacteria were detected in the epithelial cells of *Casp1* ^−/−^ mice (Fig. S7). In addition these studies confirmed better preservation of the epithelium in WT compared to *Casp1* ^−/−^ mice (Fig. S7). *Salmonella* colocalized predominantly with TROMA-1 negative cells in the mucosa and submucosa of WT and *Casp1^−/−^* mice. Most *Salmonella* colocalized with F4/80+ cells indicating that phagocytes were the main host cells for *Salmonella in vivo* (Fig. 6 C, D and Fig. S8 A, B). Quantification of *Salmonella* associated with F4/80+ cells revealed that *Casp1^−/−^* mice had significantly more infected cells and more bacteria per cell (Fig. S8 C). Electron microscopy studies confirmed this observation, and revealed that most of the bacteria in the lamina propria and submucosa of WT mice were extracellular and adjacent to cell debris, with only rare intracellular bacteria present within simple vesicles (Fig. S9). In contrast, the *Casp1* ^−/−^ lamina propria cells often harbored numerous bacteria that were frequently associated with complex and heterogeneous vacuoles, and occasionally free in the cytoplasm (Fig. S8 D and Fig. S10). These data suggest that caspase-1 controls intracellular bacterial growth *in vivo*. Immunofluorescence microscopy analysis of the MLN, spleen and liver revealed that *flgM^−^ Salmonella* were also present in F4/80+ cells in *Casp1^−/−^* mice; bacteria were not detected in these organs of WT mice (data not shown).

### The Innate Immune Receptor TLR5 Plays a Complex Role in the Attenuation of *flgM^−^Salmonella*

**Figure 7:**
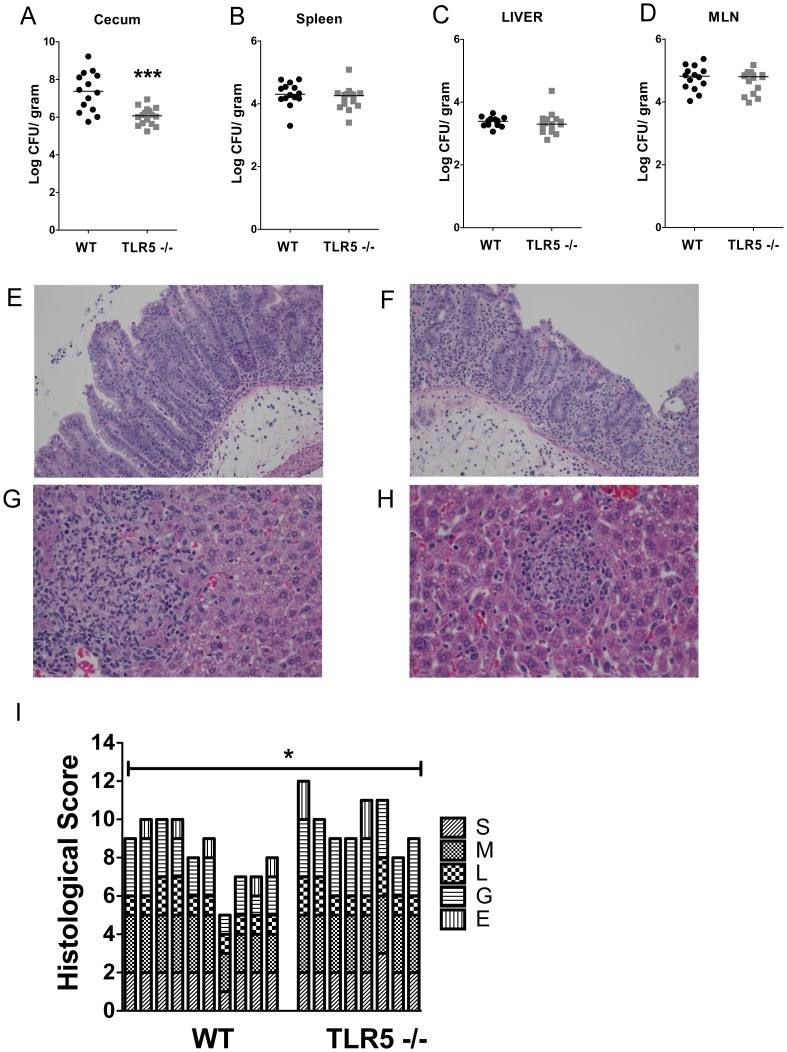
The innate immune receptor TLR5 is dispensable in the attenuation of *flgM^−^*. C57BL/6 WT (n = 14) and TLR5−/− (n = 16) mice were infected with 1000 CFU *flgM^−^ Salmonella*. Bacterial burden in the cecum (A). Spleen (B). Liver (C). MLN (D). Representative histology of the cecum for WT mice (E) or TLR5−/− (F), and liver of WT mice (G) or TLR5−/− (H). Liver images (200X magnification), cecal images (100X magnification). Figures A–D represent data from three independent experiments. Mann-Whitney test * = p<0.05. ** = p<0.01.(I) Histological scores for changes in the cecal pathology as described in Fig. 4. Figures A–D represent data from three independent experiments. Mann-Whitney test *** = p<0.001.

**Figure 8:**
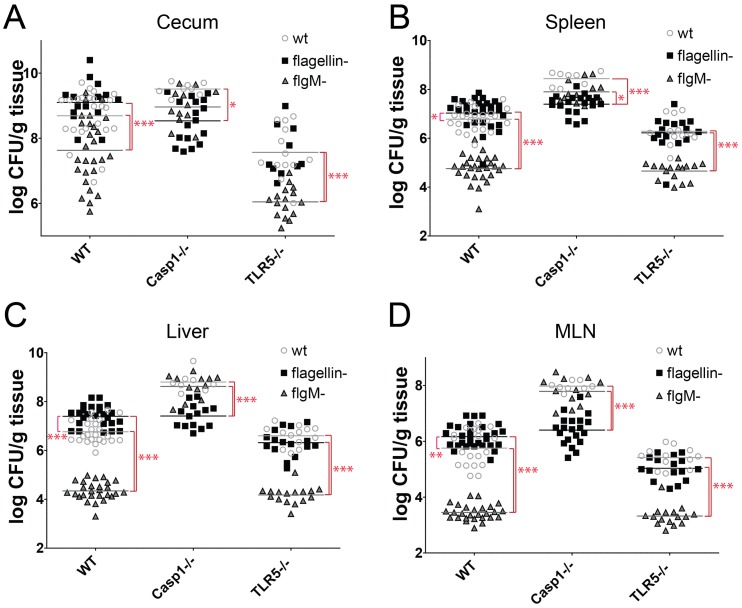
Composite analysis of Salmonella WT, flagellin- and flgM*^−^* infections in WT, caspase-1−/− and TLR5−/− mice. We combined the CFU data for all experiments using WT, flagellin deficient and *flgM^−^ S*. Typhimurium SL1344, and the data was analyzed using two-way ANOVA and Bonferroni’s multiple comparisons post tests. * = P<0.05, ** = P<0.01, and *** = P<0.001. Cecum (A), Spleen (B), Liver (C), and MLN (D).

We hypothesized that innate immune detection of flagellin by TLR5 may also contribute to the attenuated phenotype of *flgM^−^ S. typhimurium*. We first tested the role of TLR5 by infecting WT and*TLR5* ^−/−^ mice with *flgM^−^ Salmonella*. Similar to WT *Salmonella* infection (Fig. 1 E, F, G, H), bacterial burden in the spleen, liver and MLN was indistinguishable between WT and *TLR5* ^−/−^ mice (Fig. 7 A, B, C, D), indicating that the attenuation mediated by flgM deficiency is not due to flagellin detection by TLR5. Similar to WT *Salmonella* infection (Fig. 1 E), *TLR5* ^−/−^ mice also had lower bacterial burden in the cecum compared to WT mice (Fig. 8 A), consistent with our previous finding that TLR5 promotes cecal colonization of *Salmonella*. The ceca of all mice were inflamed, small and pale (Fig. S1 D). Histologically, the cecum (Fig. 7 E, F) and the liver (Fig. 7 G, H) of WT and *TLR5* ^−/−^ mice were similar, although histologic quantification showed a modest but significant increase in cecal inflammation in *TLR5* ^−/−^ mice (Fig. 7 E). Analysis of gene expression in the cecum of WT and *TLR5* ^−/−^ mice showed no significant differences in all genes tested (Fig. S2 D). Serum cytokines in WT and *TLR5* ^−/−^ showed similar levels of TNF-α, IL-1B, IL-12/23p40 in both groups; IL-6 was increased in *TLR5^−/−^* mice (Fig. S11). To confirm that TLR5 promotes cecal colonization of flgM*^−^ Salmonella* independent of *Salmonella*’s flagellin; we infected WT and *TLR5^−/−^* mice with *flgM*−/flagellin- *Salmonella*. Once again, these flagellin-deficient bacteria were even more attenuated in the *TLR5^−/−^* mice (Fig. S12), providing further evidence that the host protection provided by TLR5-deficiency is independent of flagellin expression by the pathogen, *S*. Typhimurium.

We compiled all of the CFU data from experiments that we performed using WT, TLR5−/− and Casp1−/− mice in infections with WT, flagellin-null and *flgM^−^ Salmonella* (Fig. 8). This analysis reveals that dysregulated flagellin production profoundly attenuates *Salmonella* infection in all tissues in a caspase-1 dependent manner. In the absence of caspase-1, the *flgM* deletion has essentially no phenotype, and behaves nearly identical to wt *Salmonella*. Intriguingly, both wt and *flgM^−^ Salmonella* are more virulent than flagellin- *Salmonella* in caspase-1 mice. The added virulence property that flagellin bestows upon *Salmonella* in the absence of caspase-1 may be motility, which is supported by experiments with motility and chemotaxis mutants [2], [47], or possibly some other functional interaction between flagellin and the host. The most parsimonious explanation for modest increase in virulence of flagellin- *Salmonella* relative to WT Salmonella in WT mice is that flagellin- *Salmonella* are evading inflammasome detection at the cost of losing a flagellin-dependent virulence property, such as motility. Inflammasome detection of flagellin has only been convincingly demonstrated for rodents. Because inflammasomes in humans and many other animals are incapable of flagellin detection [9], some host-restricted *Salmonella* strains, such as *S. typhi*, are not under the same selective pressures to repress flagellin expression during infection.

The composite data (Fig. 8) also illustrate that TLR5 does not contribute to the attenuation of *flgM^−^ Salmonella*, and that loss of TLR5 leads to paradoxical protection against *Salmonella* infection, which is most evident in the cecum. These experiments illuminate the complexity of host-pathogen interactions, and how the complex interplay between the host, pathogen and microbiota influence the outcome of infection. A more precise understanding of host-pathogen interactions will need to take into account the microbiota, and control for differences in microbiota that influence host-pathogen interactions.

## Discussion

In this study we demonstrate the importance of flagellin regulation by *Salmonella* and define the role of host flagellin recognition pathways in controlling systemic dissemination of bacteria, inflammation and tissue injury. Proper flagellin regulation is paramount for the infectious life cycle of *Salmonella*. *Salmonella* downregulates flagellin production in systemic sites in mice [30], [31] and disruption of flagellin regulation reduces bacterial burden and increases survival of mice [35]. Previous studies have demonstrated that deletion of *flgM*, the anti-sigma factor, leads to overproduction of flagellin and flagella *in vitro*, and attenuates *Salmonella in vivo* [35]. Our studies demonstrate that attenuation of flgM-deficient *Salmonella in vivo* is due to recognition of flagellin by the innate immune system, and predominantly by caspase-1 dependent pathways.

The role of TLR5 during *Salmonella* infection is controversial. TLR5 deficiency in mice has been associated with both increased and decreased susceptibility to WT *Salmonella* infections [23], [24], [26]. Our studies indicate that *TLR5^−/−^* mice have a complex phenotype. Similar to the findings of the Gewirtz’s and Akira’s groups [24], [25], we found that *TLR5^−/−^* mice have lower CFU in the cecum during WT *Salmonella* infection. Akira and others have demonstrated that TLR5 plays an important role in *Salmonella* trafficking by a subpopulation of dendritic cells from the cecum to the MLN, thus providing a potential explanation to our observations. More intriguingly, flagellin*^−^ Salmonella* were even more attenuated in *TLR5^−/−^* mice. When the gut is bypassed by intraperitoneal infection, we have seen no difference between WT and TLR5−/− infected with either WT or aflagellate Salmonella (M. Lai, unpublished observations). This suggests that decreased cecal colonization is responsible for the decreased colonization in systemic organs. Vijay-Kumar et al. also reported increased survival of *TLR5^−/−^* mice infected with aflagellate *Salmonella* during conventional oral infection [25] **.** These data suggest that recognition of *Salmonella’*s flagellin by TLR5 is not required for enhanced virulence of *Salmonella* in WT mice compared to *TLR5^−/−^* mice. Vijay-Kumar et al. attributed the enhanced resistance of *TLR5^−/−^* mice to an increased basal inflammatory state of *TLR5^−/−^* mice. Consistent with this hypothesis, we found that infected *TLR5^−/−^* mice had increased histologic evidence of inflammation in the ceca. However, our gene expression data showed no significant differences between infected TLR5 sufficient and deficient mice and serum cytokine measurements revealed only a modest increase in IL-6 in the TLR5−/− mice (Fig. S2 D, Fig. S11 C). In addition, we have never observed spontaneous colitis in our *TLR5^−/−^* mouse colony. Thus similar to Vijay-Kumar et al, we find that TLR5-deficiency protects mice against *Salmonella* infection, and that this protection does not require flagellin expression by *Salmonella*. *TLR5^−/−^* mice have recently been demonstrated to have an altered gut microbiota that promotes metabolic syndrome [26], and it is possible that an altered microflora or altered interactions between the host and the gut microbiota in *TLR5^−/−^* mice could establish a more effective barrier against *Salmonella* infection. A more thorough investigation of the gut microbiota in our TLR5−/− mice may help define how TLR5-deficiency is protective against Salmonella infection. Thus *Salmonella* may capitalize on TLR5-dependent host responses in the gut to gain competitive advantages over the microflora [39], [48]. Further studies are required to elucidate how TLR5 influences *Salmonella* infection in the absence of flagellin expression by *Salmonella*.

Caspase-1 protected mice against WT *Salmonella* infection (Fig. 1), consistent with previous reports [14], [15]. The deletion of flgM substantially magnified the protective capacity of caspase-1 as evidenced by 100–10,000 fold greater tissue CFU in *flgM^−^ Salmonella* infected Casp1−/− mice compared to WT mice (Fig. 5). Our results suggest that flagellin recognition by the inflammasome during WT *Salmonella* infection limits *Salmonella* spread to distant sites, and that *flgM*-dependent evasion of flagellin recognition by the inflammasome is necessary for *Salmonella* virulence.

Recently, murine TLR11 has been proposed as another flagellin receptor, and has been shown to protect mice against *Salmonella* infection and restrict *S. typhi* from growth in mice [9], [49,]. Interestingly, the virulence defect *flgM^−^ Salmonella* is completely complemented by caspase-1 deficiency, suggesting that TLR11 does not contribute to the attenuation of flgM*^−^ Salmonella*. Additional studies will be needed to clarify the role of TLR11 in this model system.

*FlgM^−^ Salmonella* infections in mice demonstrate the importance of proper flagellin regulation for virulence, systemic dissemination and inflammation. Cecal inflammation was diminished in *flgM^−^* infections compared to WT indicating a role for flagellin recognition in regulating inflammation and injury at mucosal surfaces. It is possible that excess stimulation of TLR5 or the inflammasome during mucosal infection leads to the preservation of mucosal integrity, which is further enhanced by the better control of the bacterial infection. *Casp1^−/−^* mice had more severe inflammation and injury in the cecum and higher bacterial burden in all organs indicative of caspase-1 role in protecting mucosal surfaces and containing infection. Surprisingly, *Casp1^−/−^* mice had a selective decrease in pro-inflammatory gene expression in the cecum (Fig. S2 C), suggesting that caspase-1 also promotes inflammatory responses in the cecum that may be important for limiting tissue injury. Caspase-1, as well as Nlrc6 and Asc, limit mucosal injury in dextran sodium sulfate induced colitis through the modulation of the intestinal microbiota [9]. Our data indicate that caspase-1 also limits mucosal injury in *Salmonella* infection, and this may be mediated through caspase-1-dependent control of *Salmonella* growth, through caspase-1 regulation of mucosal inflammatory responses, or a combination of these factors. Further studies will be needed to determine which inflammasomes protect the gut during *Salmonella* infection, whether alterations in the gut microbiota contribute to inflammation and injury, and whether Asc-dependent IL-1 and IL-18 are required to protect against mucosal *Salmonella* infection and preserve epithelial integrity.

Using a heterologous Spi2 promoter to drive the expression of flagellin and FliS, the flagellin chaperone, Ed Miao and colleagues also observed a strong role for caspase-1 in sensing flagellin during intraperitoneal infections [16]. In their study, neither TLR5 nor IL-1/IL-18 played a significant role in limiting *Salmonella* growth, and attenuation of their *Salmonella* strain was dependent on Nlrc4/caspase-1 and pyroptosis [16]. It is likely that pyroptosis also contributed to attenuation of flgM-deficient *Salmonella* infection, and this is supported by the electron microscopy studies demonstrating extracellular *Salmonella* adjacent to dead cells and cellular debris within the tissue of WT mice.

It has been recently demonstrated that the *Casp1* ^−/−^ mice used in this study are also deficient in Casp11 (also referred to as Casp4), and that Casp11 deficiency in the context of Casp1 deficiency is protective against *Salmonella* infection. The model predicts that Casp11 induced cell death in the absence of Casp1-dependent IL-1B and IL-18 production increases host susceptibility to *Salmonella* infection [50]. This is thought to be manifest by Casp11-mediated killing of phagocytes and release of *Salmonella*, without IL-1B mediated recruitment of neutrophils to limit *Salmonella* infection. We noted that many of the intracellular *Salmonella* seen in *Casp1^−/−^* (and *Casp11^−/−^*) mice were associated with complex phagosomes, suggesting that autophagy/xenophagy may be contributing to host defense in the combined absence of Casp1 and Casp11. Further studies will be needed to determine the interaction between these caspases and the autophagy pathway in the control of *Salmonella* infection.

Altogether, our data indicate that regulation of flagellin production by *Salmonella* is critical to evade innate immune detection, and in particular caspase-1 dependent responses. Caspase-1 is critical for controlling systemic dissemination, severe inflammation and the integrity of the mucosal barrier.

## Supporting Information

Figure S1 **Gross cecum inflammation was prevalent in all** ***Salmonella*** **infected mice.** Representative gross anatomy pictures of C57BL/6 WT mice infected with 1000 cfu WT SL1344 or *flgM^−^ Salmonella* (A). Gross anatomy pictures of C57BL/6 WT mice infected with 1000 cfu *flgM^−^* or *flgM^−/^flagellin^−^ Salmonella* (B). Gross anatomy pictures of C57BL/6 WT or caspase-1−/− mice infected with 1000 cfu *flgM^−^ Salmonella* (C). Gross anatomy pictures of C57BL/6 WT or TLR5−/− mice infected with 1000 cfu *flgM^−^ Salmonella* (D). The bar represents 1 cm.
(TIF)

Figure S2 **Flagellin and caspase-1 contribute to cytokine gene expression in the cecum.** Gene expression in the cecum measured by RT-PCR for C57BL/6 mock-infected mice, or mice infected with SL1344 WT or *flgM^−^ Salmonella* (A). Genes that are significantly induced in flgM^−^ (*) or WT and flgM^−^ (**) Salmonella infected mice compared to mock-infected mice are designated by the asterix below the x-axis (A); genes that are significantly higher in flgM^−^ infected mice are designated by the symbol (#) above the bars (A). Gene expression in the cecum measured by RT-PCR for C57BL/6 WT for flgM vs flgM/flagellin *Salmonella* infected mice (B). Gene expression in the cecum measured by RT-PCR for C57BL/6 WT or caspase-1−/−mice infected with *flgM^−^ Salmonella* (C). Gene expression in the cecum measured by RT-PCR for C57BL/6 WT or TLR5−/− mice infected with *flgM^−^ Salmonella* (D). ns = no statistical significance. Gene expression was normalized to GAPDH, and comparisons were made using the one-way ANOVA and Bonferroni’s multiple comparisons test. Genes with significant differences (P<0.05) are designated by either symbols (* or #) above the bars.
(TIF)

Figure S3 ***FlgM*** *^−^* ***Salmonella*** **overproduce flagellin** ***in vitro*** **. (**A) Western blot for flagellin from WT and *flgM^−^ S.* Typhimuirum cell pellets or supernatant; 1.5×10^8^ cell equivalents were loaded in each lane. (B) Motility of *Salmonella* measured by using a 3 g/L of agar LB plate grown at 37 C. (C) Bacterial growth in LB broth measured by OD 600. (D) *Salmonella* induced cell death in thioglycollate elicited peritoneal macrophages measured by LDH release assay. (E) TLR5 activity measured using an NF-κB luciferase reporter CHO cell assay; MTLR5-CHO cells were stimulated with 1×10^5^ heat killed cells (pellet), or 1×10^5^ cell equivalents supernatant from WT or flgM^−^ bacteria. Data are representative of two to three independent experiments.
(TIFF)

Figure S4 **Increased in inflammatory cytokines in serum of WT SL1344 infected mice.** ELISA measurement of serum cytokine for mice infected with 1000 cfu of SL1344 WT, *flgM^−^ Salmonella* or PBS (Mock): TNF-α (A), IL-1B (B), IL-6 (C), IL12/IL12 p40 (D). Figures A–D represent data from three independent experiments (WT n = 12; *flgM^−^* n = 15; mock n = 4). Statistical analysis with one-way ANOVA using the Kruskal-Wallis test and Dunn’s multiple comparisons test, * = p<0.05. *** = p<0.001.
(TIF)

Figure S5 **Increased in inflammatory cytokines in serum of Caspase-1−/− infected mice.** C57BL/6 WT or Caspase-1−/− mice were infected with 1000 cfu of *flgM^−^ Salmonella*. ELISA measurement of serum cytokine for TNF-α (A), IL-1B (B), IL-6 (C), IL-12/IL23 p40 (D). Figures A–D represent data from two independent experiments (WT n = 9; caspase-1−/− n = 8). ELISAs were not performed for all cytokines due to poor serum yield. Mann-Whitney test * = p<0.05. ** = p<0.01.
(TIF)

Figure S6 **Decreased virulence of** ***flgM^−^ Salmonella*** **is dependent on caspase-1.** *In vivo* competitive index assays where performed by infecting C57BL/6 WT (n = 5) or caspase-1−/− (n = 5) mice with an inoculum containing 500 cfu WT SL1344 and 500 cfu *flgM^−^ Salmonella*. CFU for the WT and *flgM^−^* bacteria were enumerated, and the log ratios (*flgM−/*WT) were plotted for cecum (A), liver (B), spleen (C), MLN (D). Log ratio was used to demonstrate increases or decreases of virulence between SL1344 WT and *flgM^−^Salmonella.* Mann-Whitney test * = p<0.05. ** = p<0.01.
(TIF)

Figure S7 **Ultrastructural localization of** ***flgM*** **^−^** ***Salmonella*** **in the epithelium.** Ceca from C57BL/6 WT (A,C) and Casp1−/− (B,D) mice were analyzed for electron microscopy. *Salmonella*-like bacteria were detected in the lumina for both mice (arrows, A and B), and in the cytosol of some epithelial cells in Casp1−/− mice (arrowheads, B). Rare bacteria were detected in mechanically disrupted epithelial cells of WT mice (asterix, A) and likely represent artifact. The epithelium was well preserved in the WT mice (microvilli - M, macula densa - MD, and mitochondria - M). Focally, bacteria were seen in intracellular vesicles within cells in the epithelial layer (arrowhead, D), possibly representing *Salmonella* in leukocyte vacuoles.
(TIF)

Figure S8 **Caspase-1 controls intracellular** ***Salmonella*** **.** C57BL/6 WT or Caspase-1−/− mice were infected with 1000 cfu of *flgM^−^ Salmonella* containing a stable GFP expressing plasmid. Frozen cecal tissue was stained using F4/80 antibody in WT (A) and caspase-1−/− (B). The percentage of F4/80+ cells associated with GFP+ bacteria (C) and the number of GFP+ *Salmonella* associated with each F4/80+ cell (D) were quantified for ten 40X- objective high power fields from WT and caspase-1−/− mice. Mann-Whitney test. *** = p<0.001.
(TIF)

Figure S9 **Ultrastructural localization of** ***flgM*** **^−^** ***Salmonella*** **in the lamina propria and submucosa of WT mice.** Within the lamina propria and submucosa of C57BL/6 WT mice, Salmonella-like bacteria were detected outside of cells (arrows, A) and inside of cells (arrowheads, A). Examination at higher magnification revealed that the extracellular bacteria (arrows; B,D,E) were adjacent to degenerating cells and cellular debris. The intracellular bacteria were infrequent, and present within simple vesicles contained by a single lipid bilayer (arrowheads, C).
(TIF)

Figure S10 **Ultrastructural localization of** ***flgM*** **^−^** ***Salmonella*** **in the lamina propria and submucosa of Casp1−/− mice.** Within the lamina propria and submucosa of C57BL/6 Casp1−/− mice (A–D), Salmonella-like bacteria were detected predominantly inside of cells (arrowheads), and within complex heterogenous vesicles. Some of the bacteria showed loss of integrity of the cell wall (asterix, D).
(TIF)

Figure S11 **TLR5 is dispensable in inflammatory responses against** ***flgM*** *^−^* ***Salmonella*** **.** C57BL/6 WT (n = 14) and TLR5−/− (n = 16) mice infected with 1000 cfu of *flgM^−^ Salmonella.* ELISA measurement of serum cytokine for TNF (A), IL-1B (B), IL-6 (C), IL-12 p40 (D). Figures A-D are the combined data of three independent experiments.
(TIF)

Figure S12 **TLR5 promotes cecal colonization of** ***flgM*** **^−^** ***Salmonella*** **independent of** ***Salmonella*** **flagellin.** Bacterial burden WT C57BL/6 (n = 5) and TLR5−/− (n = 4) mice infected with 1000 cfu *flgM*/flagellin*^−^ Salmonella* in the cecum (A), spleen (B), liver (C), MLN (D). Mann-Whitney test * = p<0.05.
(TIF)
