# Supplementary figures and images for: Innate Immune Detection of Flagellin Positively and Negatively Regulates Salmonella Infection
Source: PLoS One. 2013 Aug 19;8(8):e72047. doi: 10.1371/journal.pone.0072047 (PMC3747147; doi:10.1371/journal.pone.0072047)

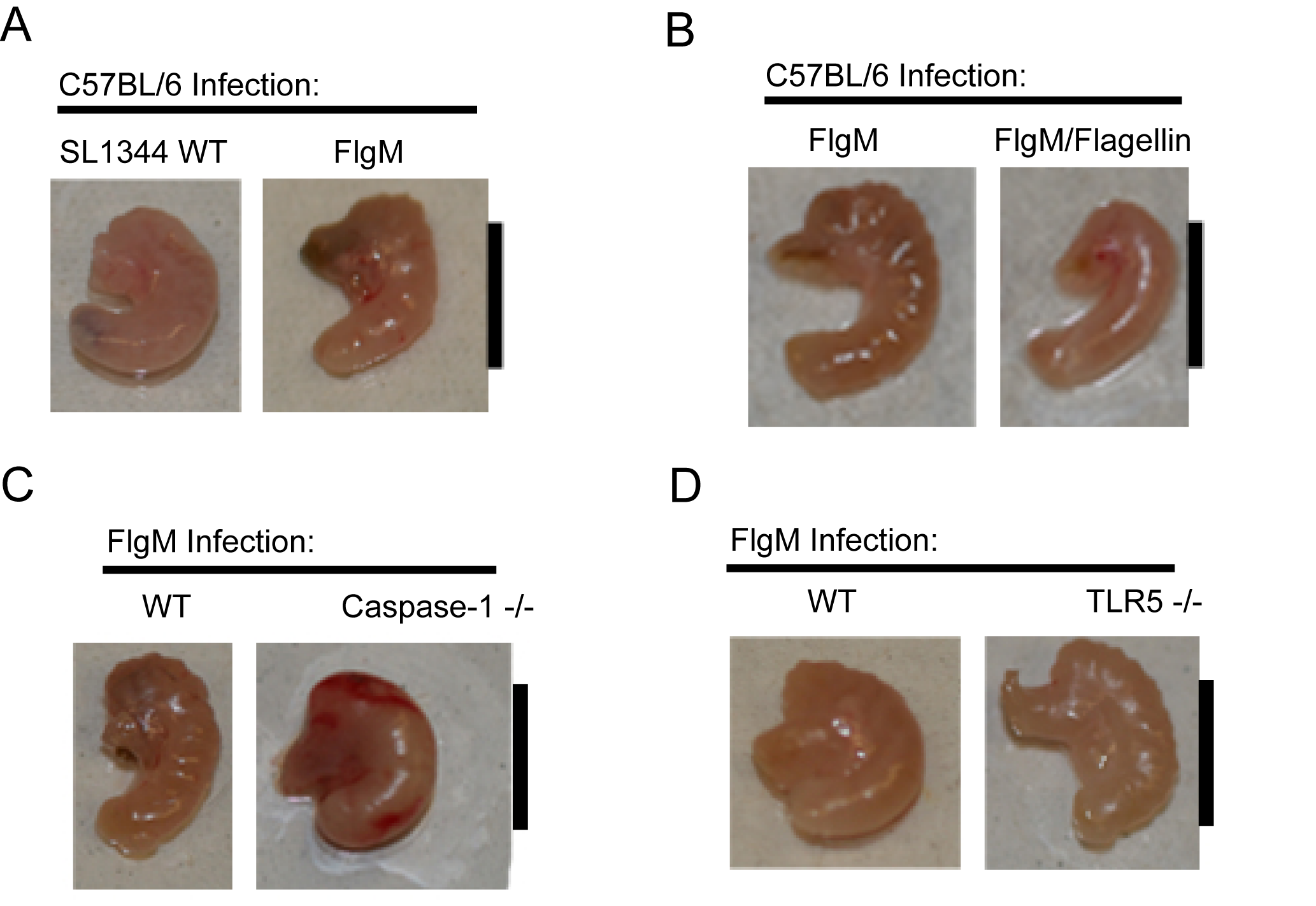

Supplement: Figure S1 — Gross cecum inflammation was prevalent in all Salmonella infected mice. Representative gross anatomy pictures of C57BL/6 WT mice infected with 1000 cfu WT SL1344 or flgM− Salmonella (A). Gross anatomy pictures of C57BL/6 WT mice infected with 1000 cfu flgM− or flgM−/flagellin− Salmonella (B). Gross anatomy pictures of C57BL/6 WT or caspase-1−/− mice infected with 1000 cfu flgM− Salmonella (C). Gross anatomy pictures of C57BL/6 WT or TLR5−/− mice infected with 1000 cfu flgM− Salmonella (D). The bar represents 1 cm. (TIF) [file pone.0072047.s001.tif]

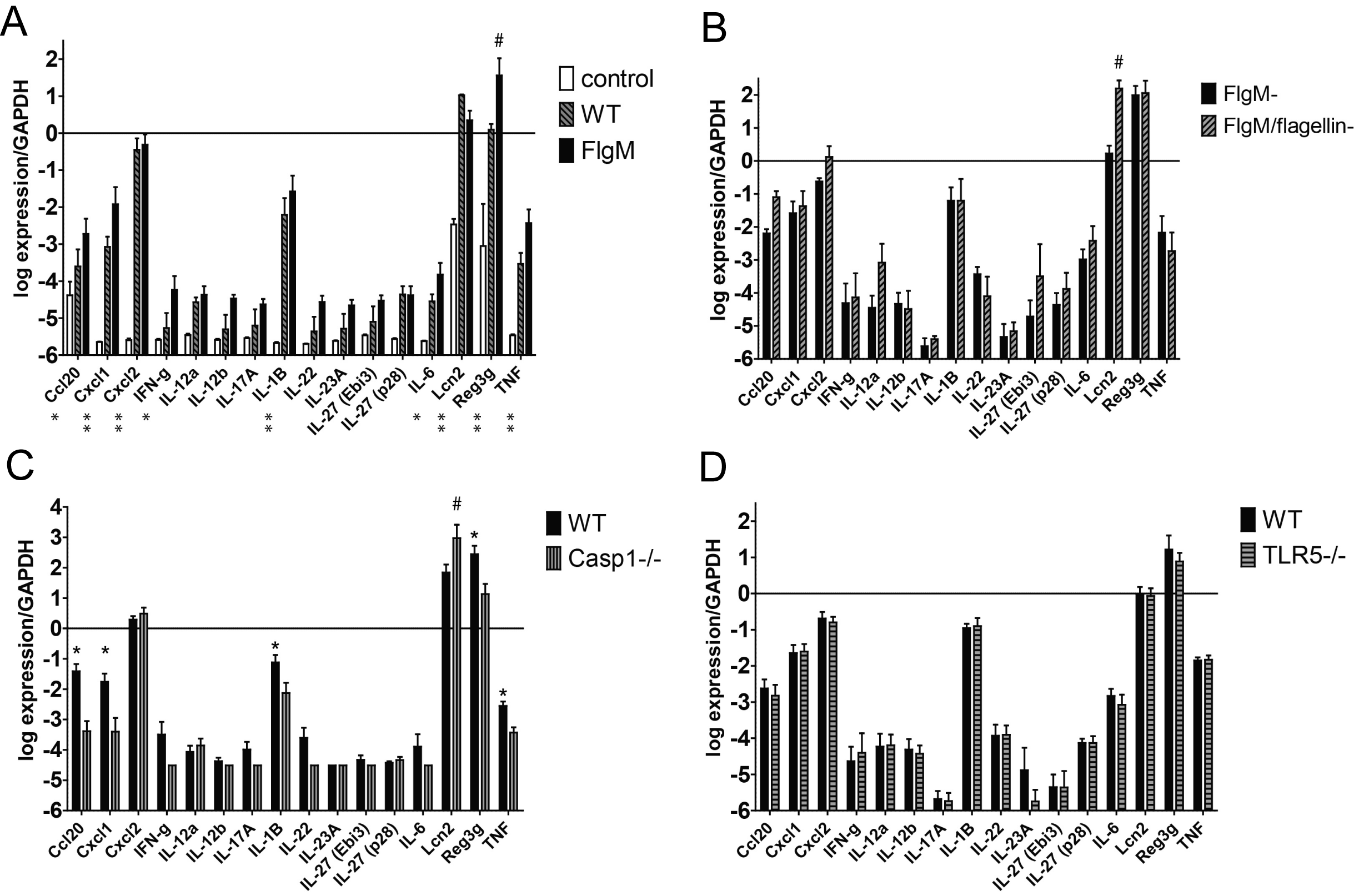

Supplement: Figure S2 — Flagellin and caspase-1 contribute to cytokine gene expression in the cecum. Gene expression in the cecum measured by RT-PCR for C57BL/6 mock-infected mice, or mice infected with SL1344 WT or flgM− Salmonella (A). Genes that are significantly induced in flgM− (*) or WT and flgM− (**) Salmonella infected mice compared to mock-infected mice are designated by the asterix below the x-axis (A); genes that are significantly higher in flgM− infected mice are designated by the symbol (#) above the bars (A). Gene expression in the cecum measured by RT-PCR for C57BL/6 WT for flgM vs flgM/flagellin Salmonella infected mice (B). Gene expression in the cecum measured by RT-PCR for C57BL/6 WT or caspase-1−/−mice infected with flgM− Salmonella (C). Gene expression in the cecum measured by RT-PCR for C57BL/6 WT or TLR5−/− mice infected with flgM− Salmonella (D). ns = no statistical significance. Gene expression was normalized to GAPDH, and comparisons were made using the one-way ANOVA and Bonferroni’s multiple comparisons test. Genes with significant differences (P<0.05) are designated by either symbols (* or #) above the bars. (TIF) [file pone.0072047.s002.tif]

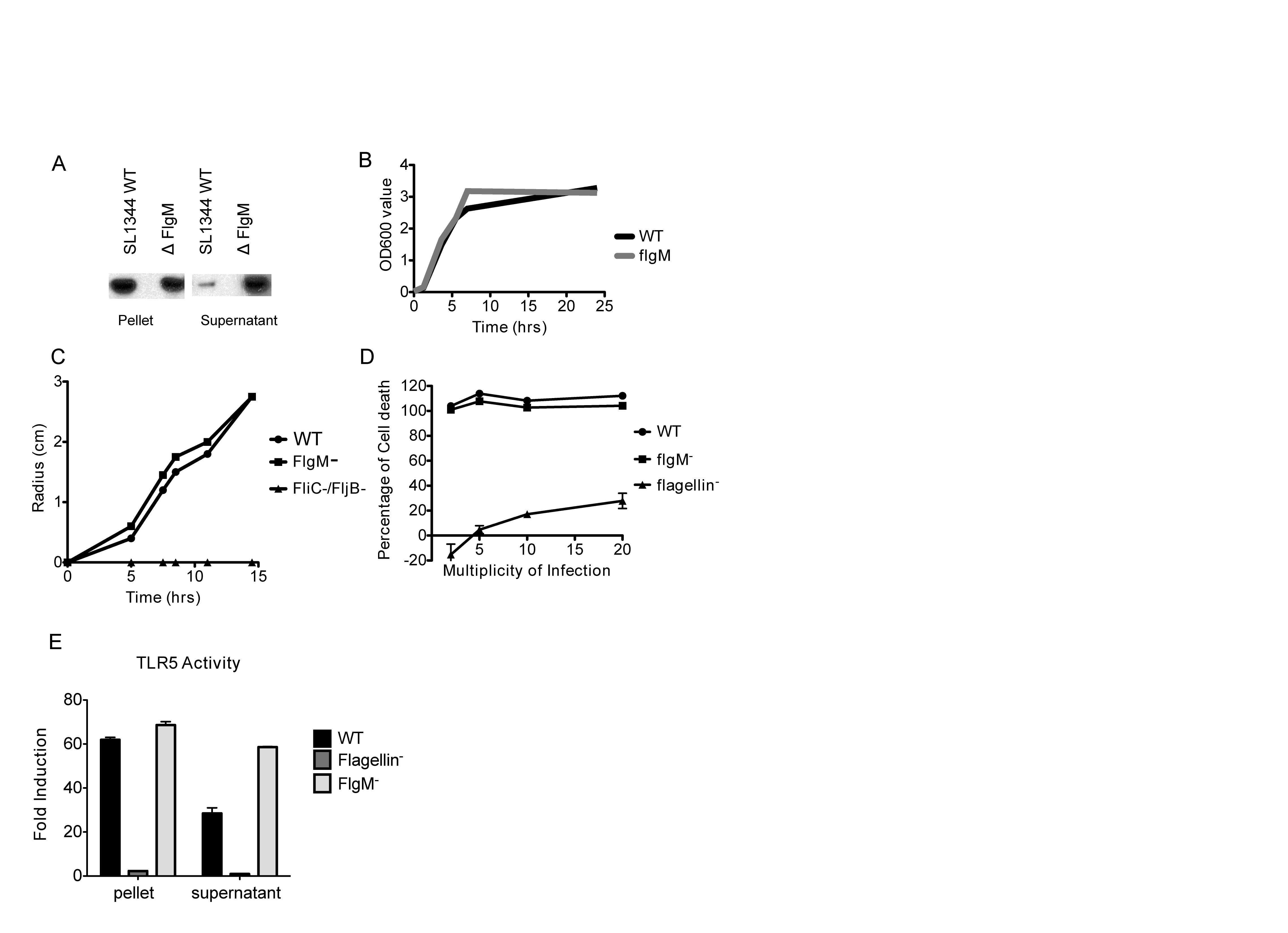

Supplement: Figure S3 — FlgM − Salmonella overproduce flagellin in vitro . (A) Western blot for flagellin from WT and flgM− S. Typhimuirum cell pellets or supernatant; 1.5×10^8^ cell equivalents were loaded in each lane. (B) Motility of Salmonella measured by using a 3 g/L of agar LB plate grown at 37 C. (C) Bacterial growth in LB broth measured by OD 600. (D) Salmonella induced cell death in thioglycollate elicited peritoneal macrophages measured by LDH release assay. (E) TLR5 activity measured using an NF-κB luciferase reporter CHO cell assay; MTLR5-CHO cells were stimulated with 1×10^5^ heat killed cells (pellet), or 1×10^5^ cell equivalents supernatant from WT or flgM− bacteria. Data are representative of two to three independent experiments. (TIFF) [file pone.0072047.s003.tiff]

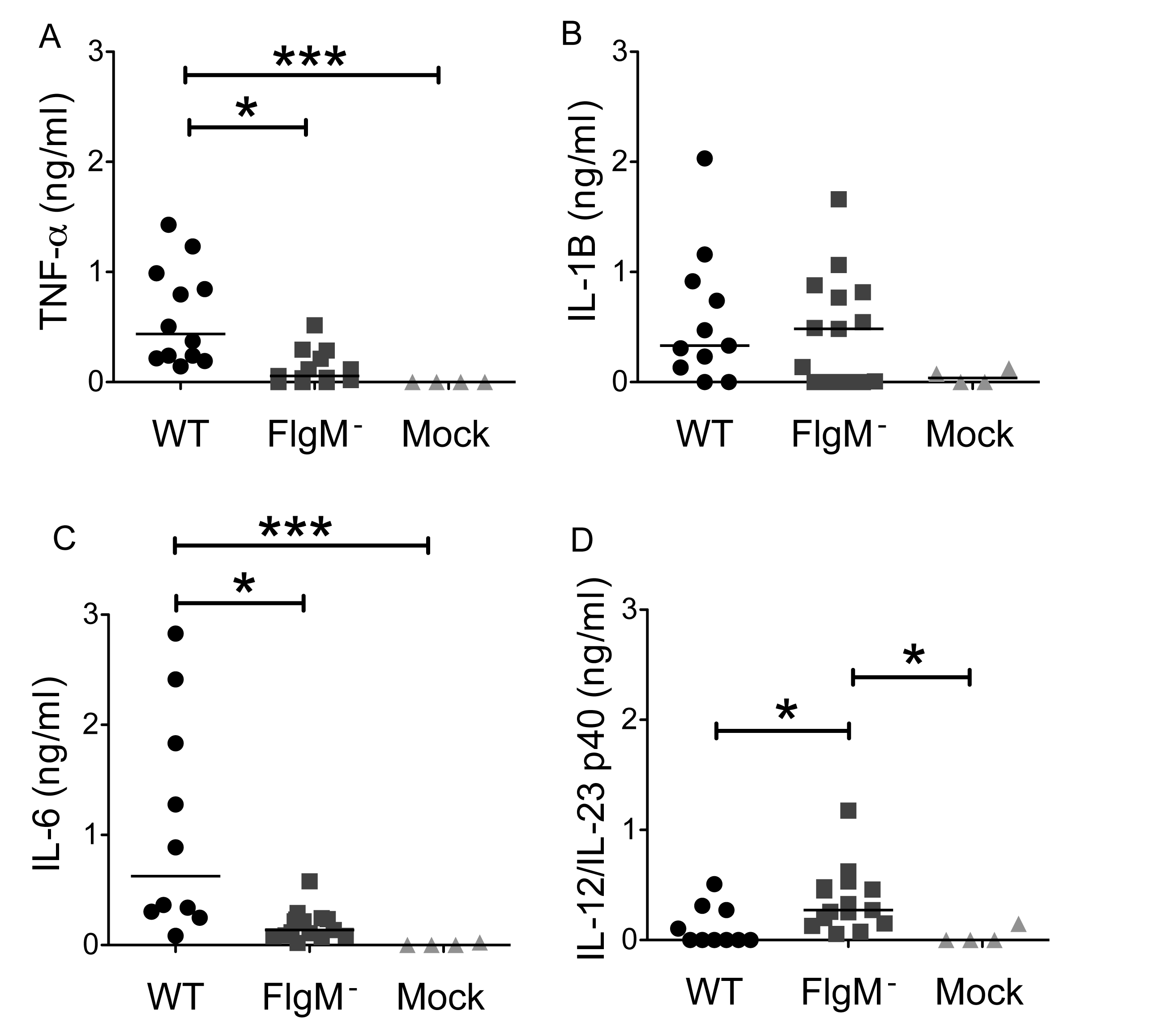

Supplement: Figure S4 — Increased in inflammatory cytokines in serum of WT SL1344 infected mice. ELISA measurement of serum cytokine for mice infected with 1000 cfu of SL1344 WT, flgM− Salmonella or PBS (Mock): TNF-α (A), IL-1B (B), IL-6 (C), IL12/IL12 p40 (D). Figures A–D represent data from three independent experiments (WT n = 12; flgM− n = 15; mock n = 4). Statistical analysis with one-way ANOVA using the Kruskal-Wallis test and Dunn’s multiple comparisons test, * = p<0.05. *** = p<0.001. (TIF) [file pone.0072047.s004.tif]

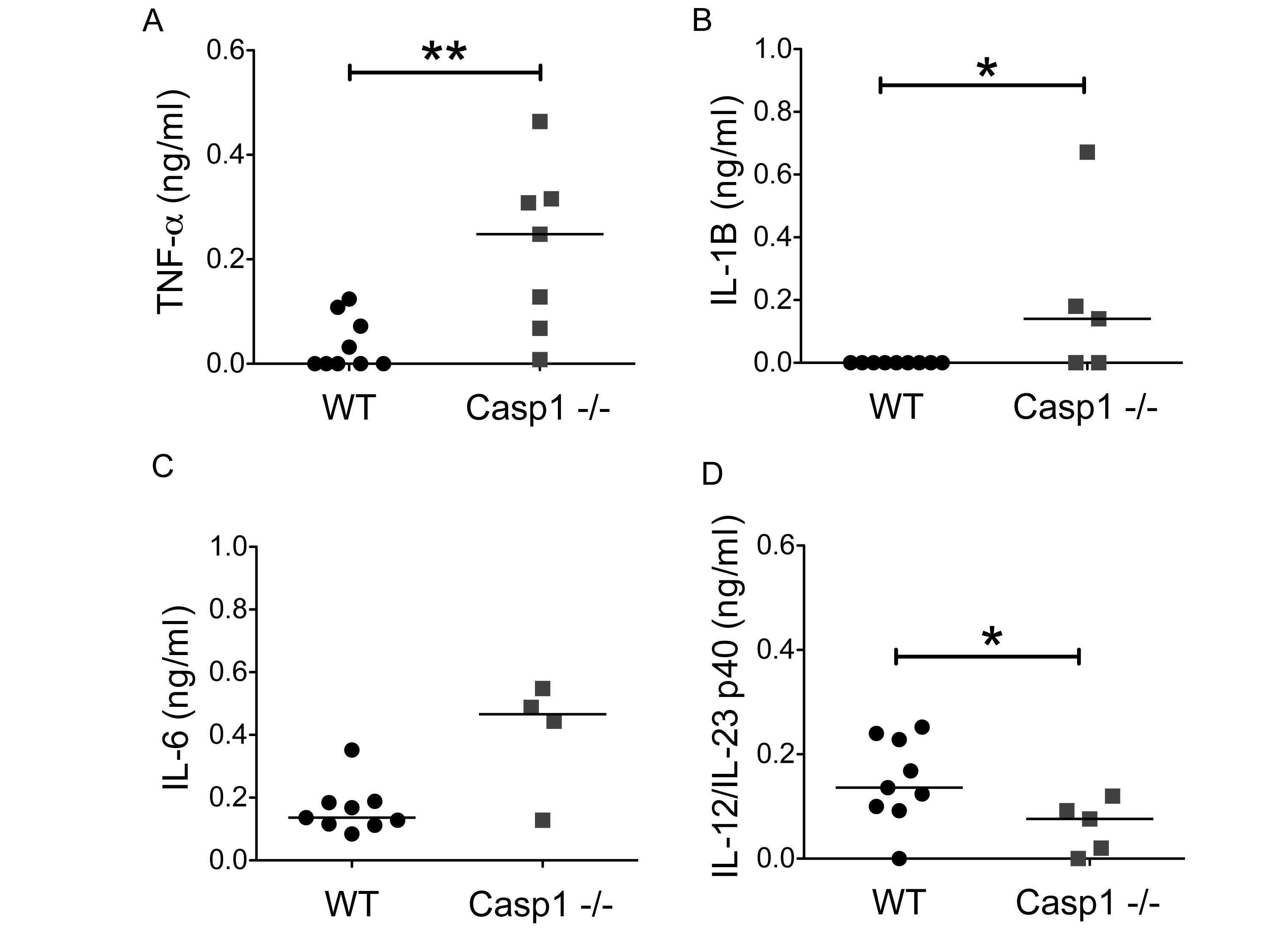

Supplement: Figure S5 — Increased in inflammatory cytokines in serum of Caspase-1−/− infected mice. C57BL/6 WT or Caspase-1−/− mice were infected with 1000 cfu of flgM− Salmonella. ELISA measurement of serum cytokine for TNF-α (A), IL-1B (B), IL-6 (C), IL-12/IL23 p40 (D). Figures A–D represent data from two independent experiments (WT n = 9; caspase-1−/− n = 8). ELISAs were not performed for all cytokines due to poor serum yield. Mann-Whitney test * = p<0.05. ** = p<0.01. (TIF) [file pone.0072047.s005.tif]

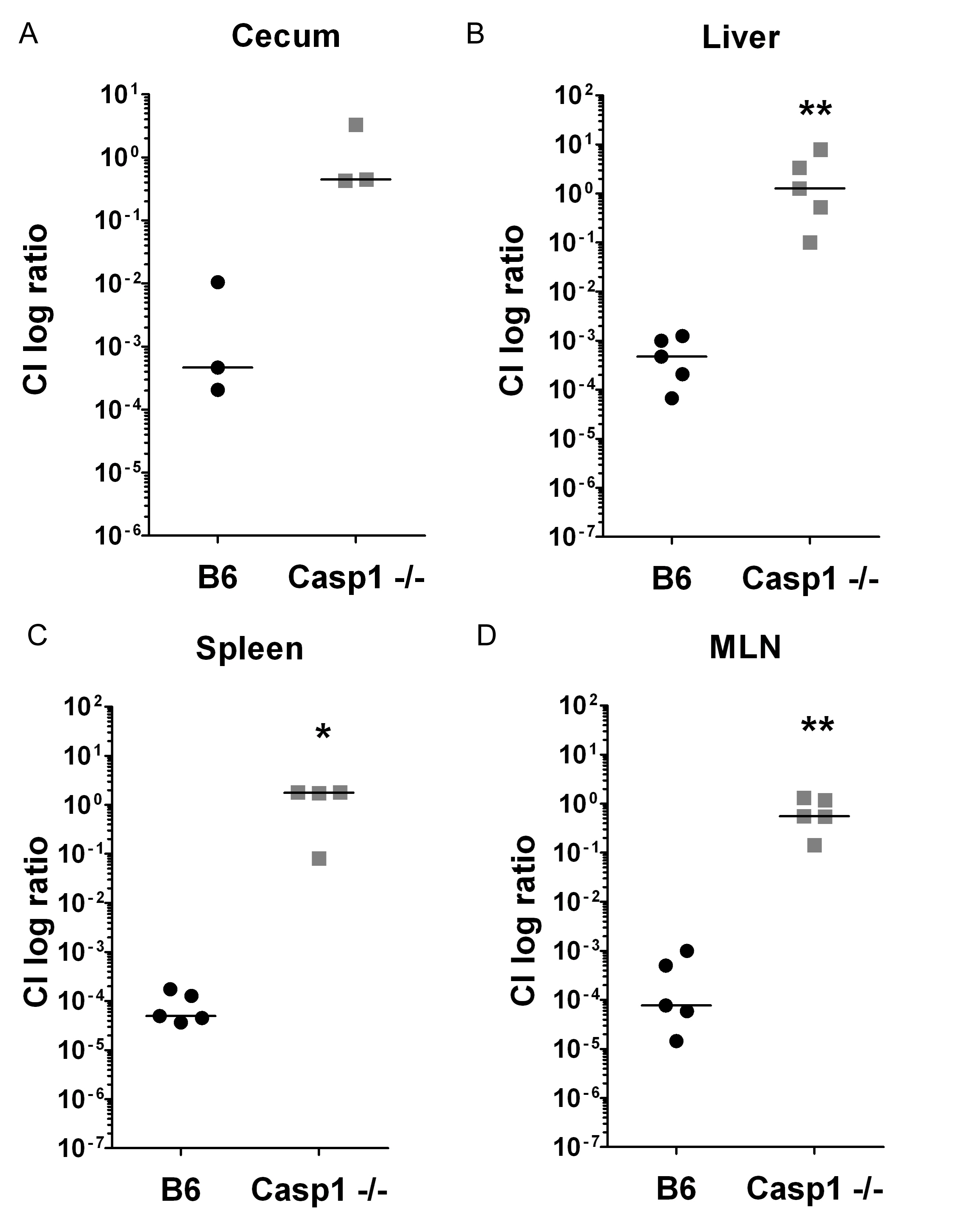

Supplement: Figure S6 — Decreased virulence of flgM− Salmonella is dependent on caspase-1. In vivo competitive index assays where performed by infecting C57BL/6 WT (n = 5) or caspase-1−/− (n = 5) mice with an inoculum containing 500 cfu WT SL1344 and 500 cfu flgM− Salmonella. CFU for the WT and flgM− bacteria were enumerated, and the log ratios (flgM−/WT) were plotted for cecum (A), liver (B), spleen (C), MLN (D). Log ratio was used to demonstrate increases or decreases of virulence between SL1344 WT and flgM−Salmonella. Mann-Whitney test * = p<0.05. ** = p<0.01. (TIF) [file pone.0072047.s006.tif]

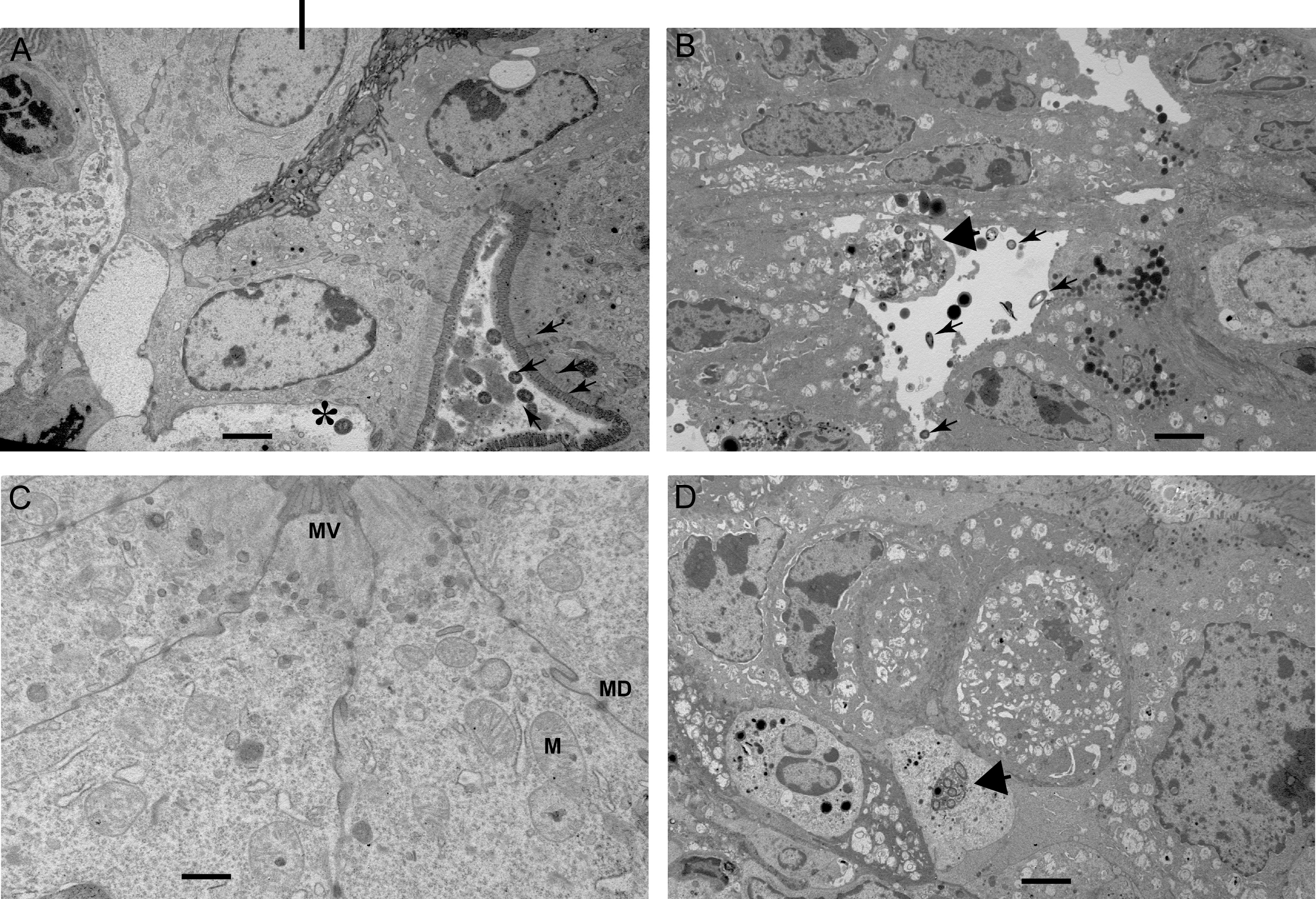

Supplement: Figure S7 — Ultrastructural localization of flgM − Salmonella in the epithelium. Ceca from C57BL/6 WT (A,C) and Casp1−/− (B,D) mice were analyzed for electron microscopy. Salmonella-like bacteria were detected in the lumina for both mice (arrows, A and B), and in the cytosol of some epithelial cells in Casp1−/− mice (arrowheads, B). Rare bacteria were detected in mechanically disrupted epithelial cells of WT mice (asterix, A) and likely represent artifact. The epithelium was well preserved in the WT mice (microvilli - M, macula densa - MD, and mitochondria - M). Focally, bacteria were seen in intracellular vesicles within cells in the epithelial layer (arrowhead, D), possibly representing Salmonella in leukocyte vacuoles. (TIF) [file pone.0072047.s007.tif]

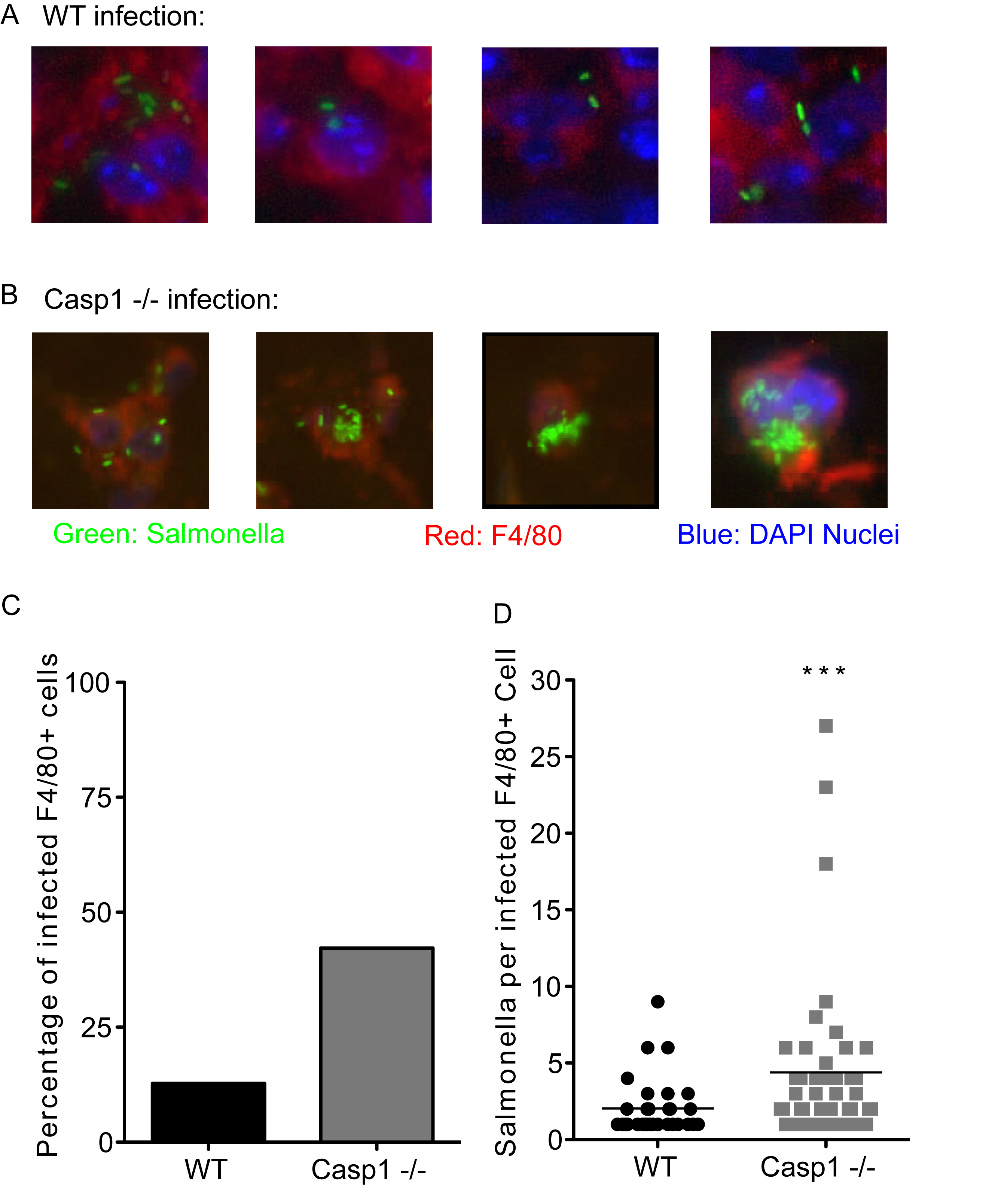

Supplement: Figure S8 — Caspase-1 controls intracellular Salmonella . C57BL/6 WT or Caspase-1−/− mice were infected with 1000 cfu of flgM− Salmonella containing a stable GFP expressing plasmid. Frozen cecal tissue was stained using F4/80 antibody in WT (A) and caspase-1−/− (B). The percentage of F4/80+ cells associated with GFP+ bacteria (C) and the number of GFP+ Salmonella associated with each F4/80+ cell (D) were quantified for ten 40X- objective high power fields from WT and caspase-1−/− mice. Mann-Whitney test. *** = p<0.001. (TIF) [file pone.0072047.s008.tif]

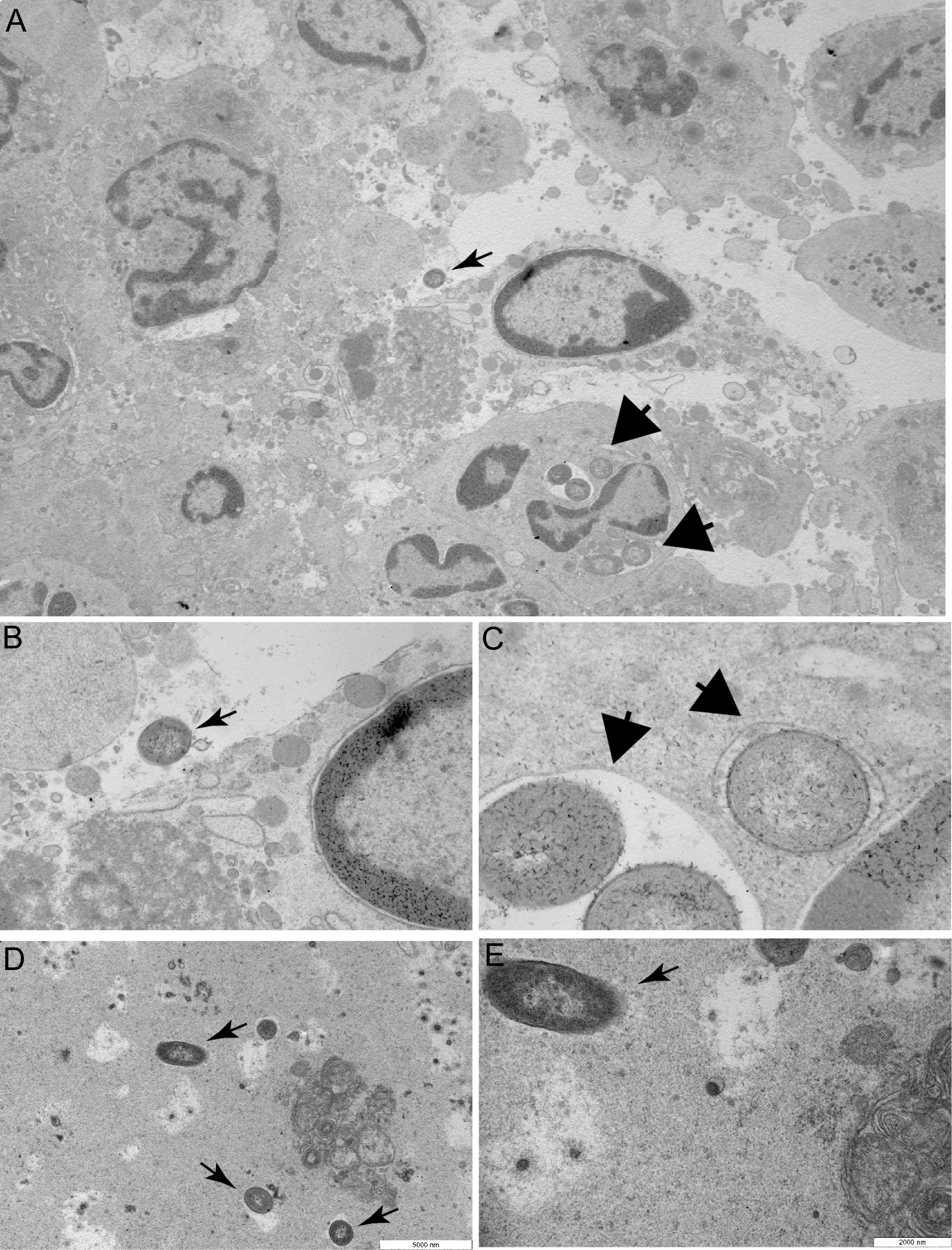

Supplement: Figure S9 — Ultrastructural localization of flgM − Salmonella in the lamina propria and submucosa of WT mice. Within the lamina propria and submucosa of C57BL/6 WT mice, Salmonella-like bacteria were detected outside of cells (arrows, A) and inside of cells (arrowheads, A). Examination at higher magnification revealed that the extracellular bacteria (arrows; B,D,E) were adjacent to degenerating cells and cellular debris. The intracellular bacteria were infrequent, and present within simple vesicles contained by a single lipid bilayer (arrowheads, C). (TIF) [file pone.0072047.s009.tif]

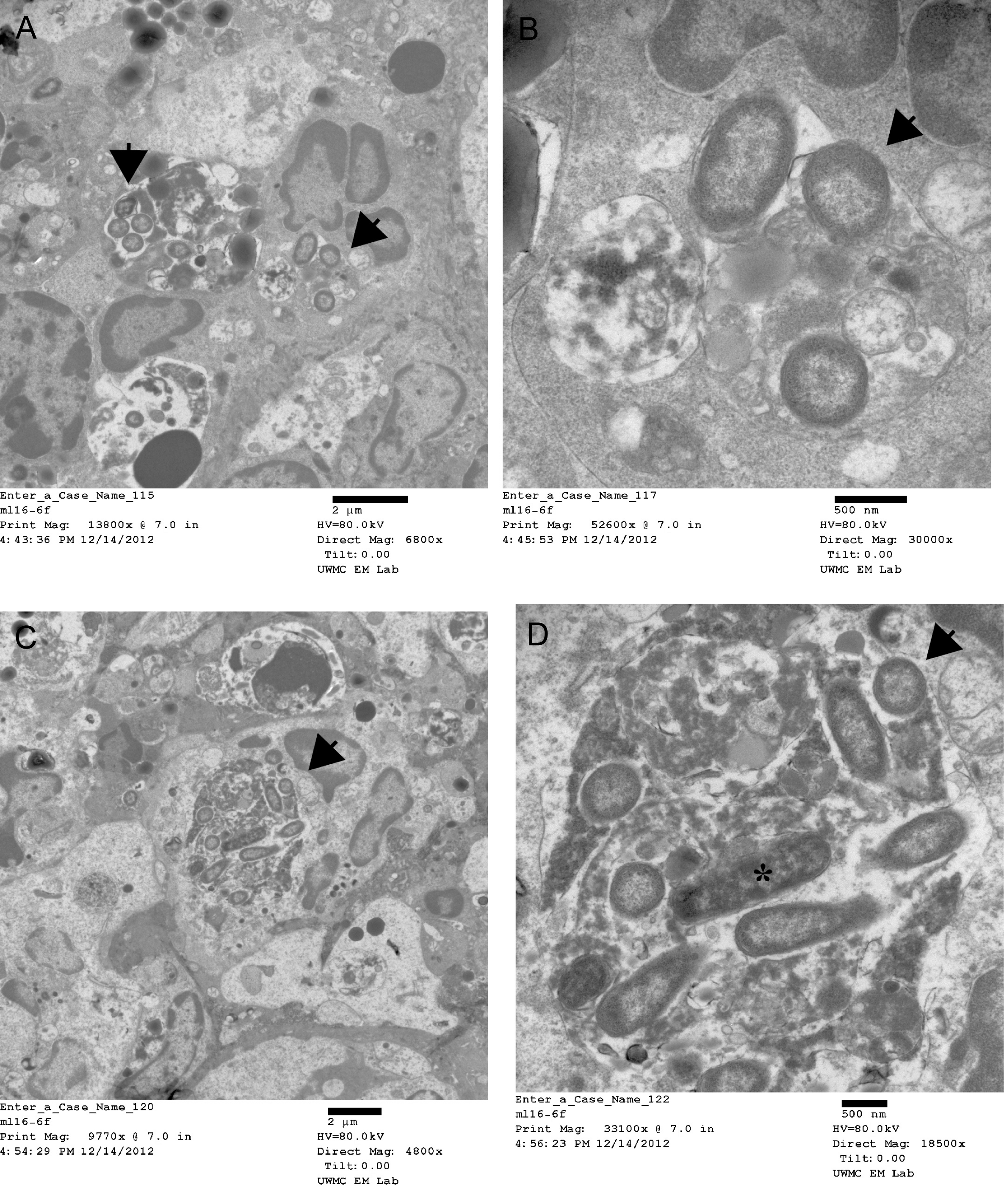

Supplement: Figure S10 — Ultrastructural localization of flgM − Salmonella in the lamina propria and submucosa of Casp1−/− mice. Within the lamina propria and submucosa of C57BL/6 Casp1−/− mice (A–D), Salmonella-like bacteria were detected predominantly inside of cells (arrowheads), and within complex heterogenous vesicles. Some of the bacteria showed loss of integrity of the cell wall (asterix, D). (TIF) [file pone.0072047.s010.tif]

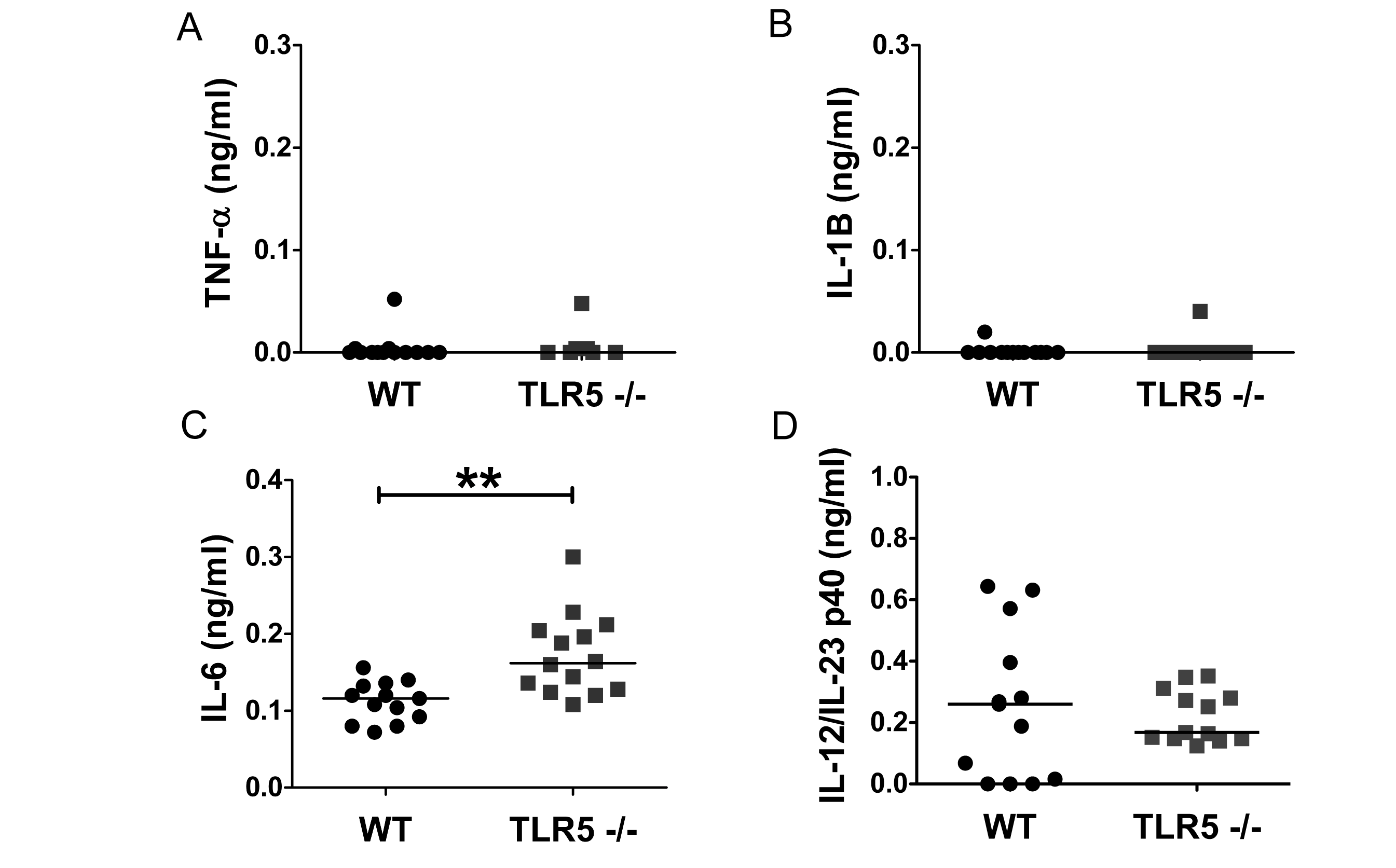

Supplement: Figure S11 — TLR5 is dispensable in inflammatory responses against flgM − Salmonella . C57BL/6 WT (n = 14) and TLR5−/− (n = 16) mice infected with 1000 cfu of flgM− Salmonella. ELISA measurement of serum cytokine for TNF (A), IL-1B (B), IL-6 (C), IL-12 p40 (D). Figures A-D are the combined data of three independent experiments. (TIF) [file pone.0072047.s011.tif]

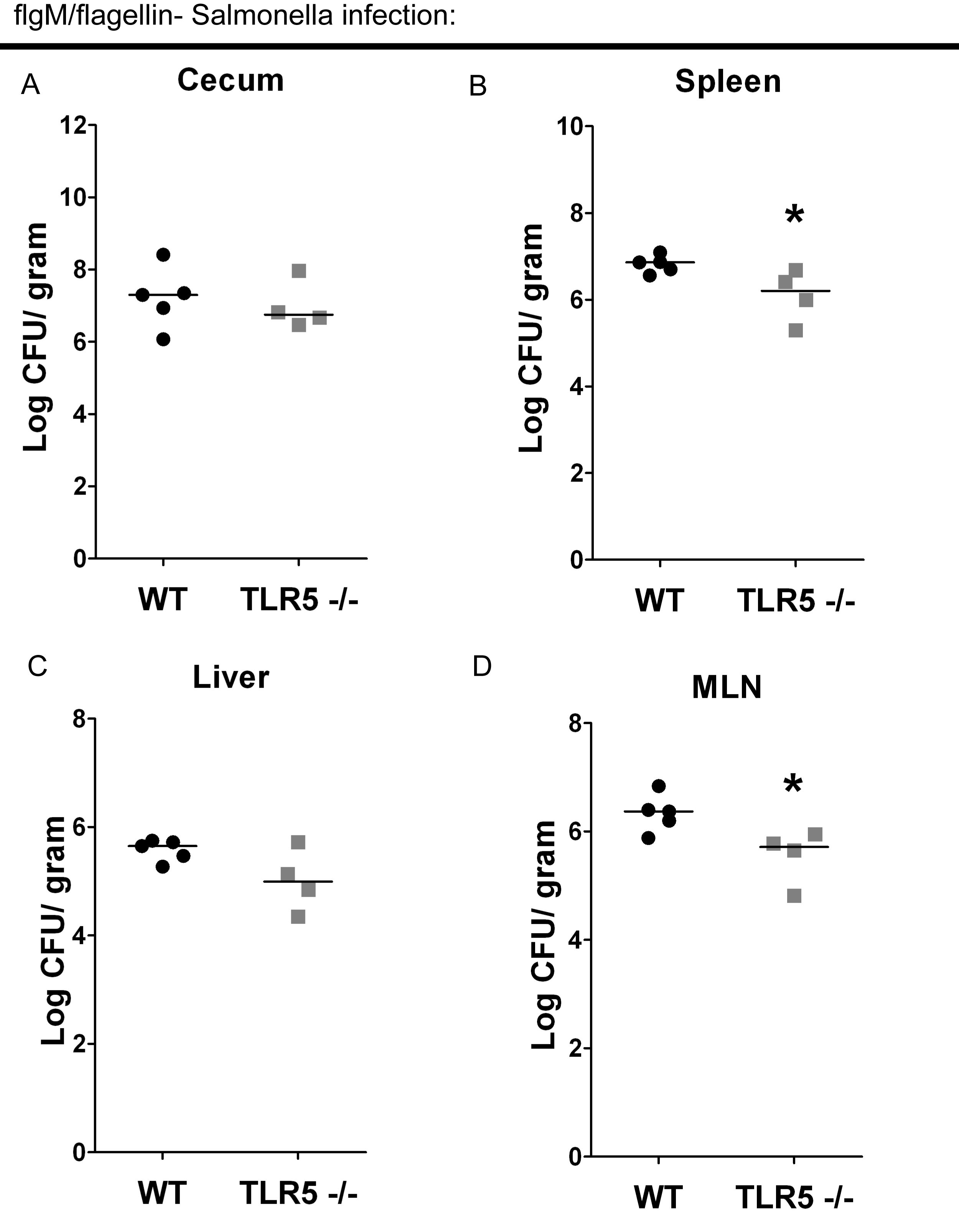

Supplement: Figure S12 — TLR5 promotes cecal colonization of flgM − Salmonella independent of Salmonella flagellin. Bacterial burden WT C57BL/6 (n = 5) and TLR5−/− (n = 4) mice infected with 1000 cfu flgM/flagellin− Salmonella in the cecum (A), spleen (B), liver (C), MLN (D). Mann-Whitney test * = p<0.05. (TIF) [file pone.0072047.s012.tif]
